# Biological research on mental pain, social pain and other pains not primarily felt in the body: methodological systematic review

**DOI:** 10.1192/bjp.2024.292

**Published:** 2025-10

**Authors:** Etienne K. Duranté, Alexandre Ribeiro, Lucie Gaspard-Boulinc, Isabelle Boutron, Chantal Henry, Anne-Cecile Petit, Josselin Houenou, Cedric Lemogne, Astrid Chevance

**Affiliations:** Université de Paris Cité and Université Sorbonne Paris Nord, Inserm, INRAE, Center for Research in Epidemiology and StatisticS (CRESS), Paris, France; Centre d′Épidémiologie Clinique, AP-HP, Hôpital Hôtel Dieu, Paris, France; Institut Curie, PSL Research University, Paris, France; Department of Psychiatry, Service Hospitalo-Universitaire, GHU Paris Psychiatrie & Neurosciences, Paris, France; Université Paris-Cité, Paris, France; Institut Pasteur, Université Paris Cité, Paris, France; NeuroSpin, CEA, Université Paris-Saclay, Gif-sur Yvette, France; APHP, CHU Mondor, DMU IMPACT, INSERM U955 Team ‘Neuropsychiatrie Translationnelle’, IMRB, Université Paris Est Créteil, Créteil, France; Department of Psychiatry, AP-HP, Hôpital Hôtel Dieu, Paris, France

**Keywords:** Cyberball, mental pain, social pain, suffering, suicide

## Abstract

**Background:**

Researchers explore the biology of painful experiences not primarily felt in the body (‘non-physical pain’), sometimes referred to as mental, social or emotional pain. A critical challenge lies in how to operationalise this subjective experience for biological research, a crucial process for translating findings into clinical practice.

**Aims:**

To map studies investigating biological features of non-physical pain, focusing on their conceptual features (i.e. terms and definitions of non-physical pain) and methodological characteristics (e.g. experimental paradigms and measures).

**Method:**

This methodological systematic review searched reports of primary research on the biological features of non-physical pain across Embase, MEDLINE and Web of Science. Using a meta-research approach, we synthetised results on terms, definitions, populations, experimental paradigms, confounders, measures of non-physical pain and investigation methods (e.g. functional magnetic resonance imaging).

**Results:**

We identified 92 human studies, involving 7778 participants. Overall, 59.1% of the studies did not report any definition of non-physical pain, and 82% of studies did not use a specific measure. Regarding the possibility of translating results to clinical settings, most of the human studies involved only healthy participants (71.7%) and the seven different experimental paradigms used to induce non-physical pain had unknown external validity. Confounders were not considered by 32.4% of the experimental studies. Animal studies were rare, with only four rodent studies.

**Conclusions:**

Biomedical studies of non-physical pain use heterogeneous concepts with unclear overlaps and methods with unknown external validity. As has been done for physical pain, priority actions include establishing an agreed definition and measurement of non-physical pain and developing experimental paradigms with good external validity.

A range of different terms are used by researchers to refer to experiences described as painful yet without being primarily felt in the body, such as ‘mental pain’^1^, ‘social pain’^2,3^, ‘emotional pain’^1^, ‘psychological pain’^1,4,5^ and ‘psychache’^6^.^,^ For instance, Shneidman, after reading hundreds of suicide letters, defined ‘psychache’ as a pain ‘felt in the mind’ caused by deprived needs; Meerwijk and Weiss defined ‘psychological pain’ as a ‘lasting unsustainable feeling’ and developed a self-reported measurement instrument in the form of a questionnaire. In the field of neuroscience, Eisenberger et al forged in 2003 the term ‘social pain, further defined in 2004 as describing ‘the pain experienced upon social injury when social relationships are threatened, damaged or lost’.^3,7^ Although physical pain might be associated with such experiences, it is secondary to the painful experience that is primarily felt in the mind. Hence, these pains are distinct from psychogenic pain, which is a physical pain (primarily felt in the body) caused by psychological events. In this body of research, the adjectives ‘mental’, ‘psychological’, ‘emotional’, ‘social’, etc. associated with the word ‘pain’ characterise both phenomenal aspects (i.e. where it is painful) and aetiopathogenesis (i.e. what caused the pain). For clarity and brevity in the present work, we will subsume all these different terms used by researchers to refer to pain not primarily felt in the body (e.g. mental pain, psychological pain, psychache, social pain) with the portmanteau word ‘non-physical pain’.

## Historical background

In psychiatry, notions of pain not primarily felt in the body trace back to the 19th century in Europe.^8^ Although some psychiatrists of that time defined ‘mental pain’ as a core symptom of melancholia (e.g. Maudsley, Griesinger, Séglas), others identified it as a common symptom in mental disorders allowing separation of normal from pathological experiences (e.g. Guislain).^8^ This has a posterity in the 20th century, in particular in the description of melancholia in Ey et al’s classic textbook of psychiatry, first published in 1960.^9^ More recently, clinical research on non-physical pain has focused on the link with suicide.^5,10,11^ Following Shneidman’s work, different measurement scales for non-physical pain have been developed for the purpose of clinical research in psychiatry and suicidology and have led to two main results: non-physical pain might be a symptom associated with several mental disorders (e.g. depressive disorders, borderline personality disorder, obsessive–compulsive disorder, schizophrenia) and it can be a predictor of suicide.^1,10,12–16^

One might argue that in the terms ‘mental pain’, ‘psychological pain’, etc., pain is to be understood metaphorically. However, researchers have investigated whether they share biological features with physical pain.^2,3,16–19^ Conducting research on the biology of non-physical pain raises many challenges, such as conceptualising a subjective experience for the purpose of experimental research, developing models of non-physical pain in humans – and non-humans – and the question of how to measure this pain.^1,20,21^ A critical challenge lies in how to effectively operationalise this deeply subjective human experience for biological research, a process crucial for translating findings into clinical practice.

## The current study

This study adopts an epistemological approach to describe what concepts and methods have been used in contemporary biological research to investigate non-physical pain. Hence, the objective of this study is to comprehensively map studies investigating biological features of non-physical pain, focusing on their conceptual features (i.e. terms and definitions) and methodological characteristics (e.g. experimental paradigms, measures, controls). Research mappings are methodological overviews that allow for an assessment of a research field by identifying key concepts, methods, studied populations, etc.^22–26^ They have been shown to help researchers understand the landscape of existing research, identify gaps and highlight trends or patterns in a particular area. In doing so, they provide material to think about how research is conducted and propose ways forward. Methodological appraisal of biological investigations of non-physical pain is scarce and non-systematic.^17,27–29^ Hence, inquiring about the concepts and methods mobilised by contemporary researchers investigating the biological features of non-physical pain might have critical influences on the broader understanding of pain, suffering and the potential development of treatment. At this point, we acknowledge that ‘non-physical pain’ is neither a concept nor a construct but a practical category referring to pain not primarily felt in the body. Investigating whether non-physical pain is a real phenomenon or naturalistic entity is beyond the scope of this research mapping study.

## Method

We conducted a methodological systematic review of published original studies exploring the biological research of non-physical pain, compliant with the PRISMA guidelines.^30^ The protocol of the study is available on Zenodo (https://zenodo.org/doi/10.5281/zenodo.6984681).

### Eligibility criteria

We included published reports of primary research studies that investigated at least one biological feature (hereafter, biomarker) hypothetically associated with non-physical pain, the subjective experience of pain not primarily felt in the body. In the context of our study, we defined a biomarker of non-physical pain as any ‘defined characteristic that is measured as an indicator of normal biological processes, pathogenic processes, or biological responses to an exposure’ at any level (genetic, molecular, cellular, neural circuits, and physiology or pathophysiology) for which the authors of the study hypothesised an involvement in non-physical pain.^31^

We excluded conference talks, posters, dissertations, textbook chapters and all secondary research (i.e. evidence synthesis such as narrative review, systematic review, meta-analysis). We excluded studies investigating close concepts of non-physical pain yet not explicitly mentioning a reference to pain in their definition (e.g. stress, negative emotions). If a published report included several studies, we applied the eligibility criteria at the study level, and not only at the report level. For instance, DeWall et al^32^ included two different studies, only one of which met our eligibility criteria.

In articles reporting systematic secondary research (i.e. systematic evidence synthesis such as systematic review, meta-analysis) identified through our search strategy, we screened their reference lists in search of additional primary research that might have been overlooked by our search strategy.

### Information sources and search strategy

In compliance with Cochrane guidelines, we contacted an information specialist of the Medical Library of Paris Cité University, who checked the relevance of the databases for the topic, the list of keywords and the search queries for each database.^33^

We searched three databases, which are the most relevant for biology-related publications – MEDLINE, Embase and Web of Science – from their date of inception to 6 November 2023.

Based on our previous systematic review of clinical studies on non-physical pain, we anticipated heterogeneity in the terms and definitions of non-physical pain.^1^ We therefore developed a pilot iterative search strategy based on the inclusion of different synonyms, using a bibliographic visualising tool showing the overlap between keywords, and comparing different combinations of keywords.^33–36^ Starting from the keywords used in our previous systematic review, which used strict synonyms ‘mental pain’, ‘psychological pain’, ‘psychache’ and ‘psychic pain’,^1^ we added ‘psychalgia’, a strict historical synonym.^8^ This research retrieved only around 30 papers addressing biological features. We therefore decided to expand the keywords by asking experts in cognitive neuroscience, who recommended that we add ‘social pain’ and ‘social distress’ because they are considered as non-physical pain in this field.^2,^ ^37^ We also included ‘emotional pain’, which was formalised in psychological science by Bolger and has been used as a synonym of non-physical pain in various works.^38–41^ Also, we included ‘spiritual pain’ and ‘soul pain’, coined by Saunders as one of the dimensions of total pain.^42–48^ Finally, to allow for a broader search, we included the keywords ‘mental suffering’, ‘psychological suffering’, ‘emotional suffering’, ‘painful feelings’ and ‘hurt feelings’, as our pilot search strategy showed that they were used as synonyms in biological publications. We also investigated the notion of ‘psychological distress’ through MEDLINE but an examination of the context of the use of these words in a sample of approximately 200 articles allowed us to conclude that most of the time it is used as a synonym for symptoms of anxiety or depression experienced in response to adverse events, which are outside the scope of this review. We did not include ‘social rejection’ or ‘social exclusion’ in our search equations, as these two terms refer to experimental paradigms or objective facts, and not to the subjective experience of non-physical pain. To evaluate the validity of this search strategy, we used a programming tool based on text mining and network co-occurrence with different bibliographic files from different keywords combinations on two databases (MEDLINE and Web of Science) and found keywords similar to those identified with the previous methods.^49^

The final search query consisted of 15 terms: ‘mental pain’, ‘psychological pain’, ‘psychache’, ‘psychalgia’, ‘psychic pain’, ‘emotional pain’, ‘spiritual pain’, ‘soul pain’, ‘mental suffering’, ‘psychological suffering’, ‘emotional suffering’, ‘painful feelings’, ‘hurt feelings’, ‘social pain’ and ‘social distress’. Search equations for each database are displayed in Supplementary material 1, available at https://doi.org/10.1192/bjp.2024.292.

### Selection of the relevant articles

After removal of duplicates, two investigators (E.K.D. with either A.R. or A.C.) independently screened the articles by title and abstract using the Covidence systematic review software.^50^ The eligibility criteria were refined during the selection of the first 5% of the sample. Disagreements were resolved by discussion and consensus. The same procedure was applied to the screening of full-text articles. We also screened the references included in systematic reviews and meta-analyses identified through our search strategy.

### Data extraction

#### Procedure

A data charting form listing all extracted data was jointly developed by E.K.D., L.G.-B. and A.C. The form was tested and refined by the three authors on 5% of the sample. One investigator (E.K.D. or A.R.) extracted the information from all full text. A second investigator (A.C.) checked 70% of the extracted data.

#### Characteristics of the studies

For each included published report, we extracted the journal name, year of publication, author(s), title and continent of the first author’s institution. We extracted the type of study subjects/participants (rodents or humans), the design (observational or experimental), the population (setting, health status, sample size). We also extracted whether one of the aims of the study was to investigate the shared physiological basis of non-physical pain with physical pain. Finally, we extracted the name of each biomarker that the authors reported to have measured in association with non-physical pain, as reported in the methods and/or results section of their study, as well as the investigation methods used (e.g. functional magnetic resonance imaging (fMRI), electroencephalogram (EEG)). For studies using fMRI, we investigated whether they used whole brain analysis and/or analyses on specified regions of interest (ROIs) and/or functional connectivity analysis (with or without connectivity graphs).

#### Conceptual features

We extracted the terms used to refer to non-physical pain (pain not primarily felt in the body). We also extracted the definition corresponding to each term, if any, and potential associated references. By ‘definition’, we broadly understand any statement describing the non-physical pain either by its nature, attributes and/or function or by examples. For instance, if an article reported a definition of social pain by Eisenberger and a definition of psychological pain by Shneidman, we extracted both, as the author reported them, and their corresponding references.

#### Methodological characteristics

For observational studies, we extracted the exposure to non-physical pain (for instance some authors considered depression or suicidality as an exposure to non-physical pain) and the design regarding measurement at multiple time points (e.g. prospective cohort) or a single time point (e.g. cross-sectional design). For experimental studies, we extracted the name and description of the experimental paradigm used to induce or mimic non-physical pain and any reference to an external validation study. We extracted whether a control for potential confounders was used in the paradigm to assess the specificity of the biomarker regarding non-physical pain (e.g. if the effect of surprise linked to the unannounced induction of pain was considered). As an important component of the paradigm, we specifically extracted whether the duration of the induction of the non-physical pain was reported. Finally, for both observational and experimental studies, we extracted the name of any instrument used to measure non-physical pain. For experimental studies, we also extracted whether the timeline to the measurement of non-physical pain after induction/mimicking was reported.

### Analysis and synthesis of results

Regarding the analysis of conceptual features, we calculated the number of different terms used per study. Two investigators (E.K.D. and A.C.) performed an inductive thematic qualitative content analysis of the definitions of the different non-physical pain retrieved.^51^ For each term (e.g. social pain, mental pain), we then proposed a typology of their corresponding definitions based on the most frequent themes retrieved (themes cited by more than 10% of the definitions attached to the corresponding term).

Regarding the analysis of methodological characteristics, one researcher (E.K.D.) inductively classified the experimental paradigms depending on the nature of the task inducing non-physical pain and calculated the frequency and percentage of use across the sample. The researcher classified all unique biomarkers in overarching categories using biological and semantic considerations. This classification was checked by another researcher (L.G.-B. or J.H.). One researcher (E.K.D.) classified the investigation methods depending on their technical nature and their proximity to other investigation methods (e.g. ‘SNP genotyping method via MALDI-TOF’ and ‘SNP analysis’ were classified as ‘SNP genotyping’). This classification was checked by another researcher (L.G.-B. or J.H.).

We then analysed methodological patterns of investigations of biomarkers across experimental studies by identifying each unique combination of an experimental paradigm plus a categorised or uncategorised investigation method plus a biomarker category. We used a Sankey diagram to visualise these methodological patterns, created with the *ggalluvial* library.^52^

Regarding measures of non-physical pain, we categorised each of them as a self-reported or an observer-reported measure. Using the validation paper reported by the authors, we extracted the construct it aimed to measure (e.g. the Positive and Negative Affect Scale measures the presence of positive and negative emotions).

## Results

**Fig. 1:**
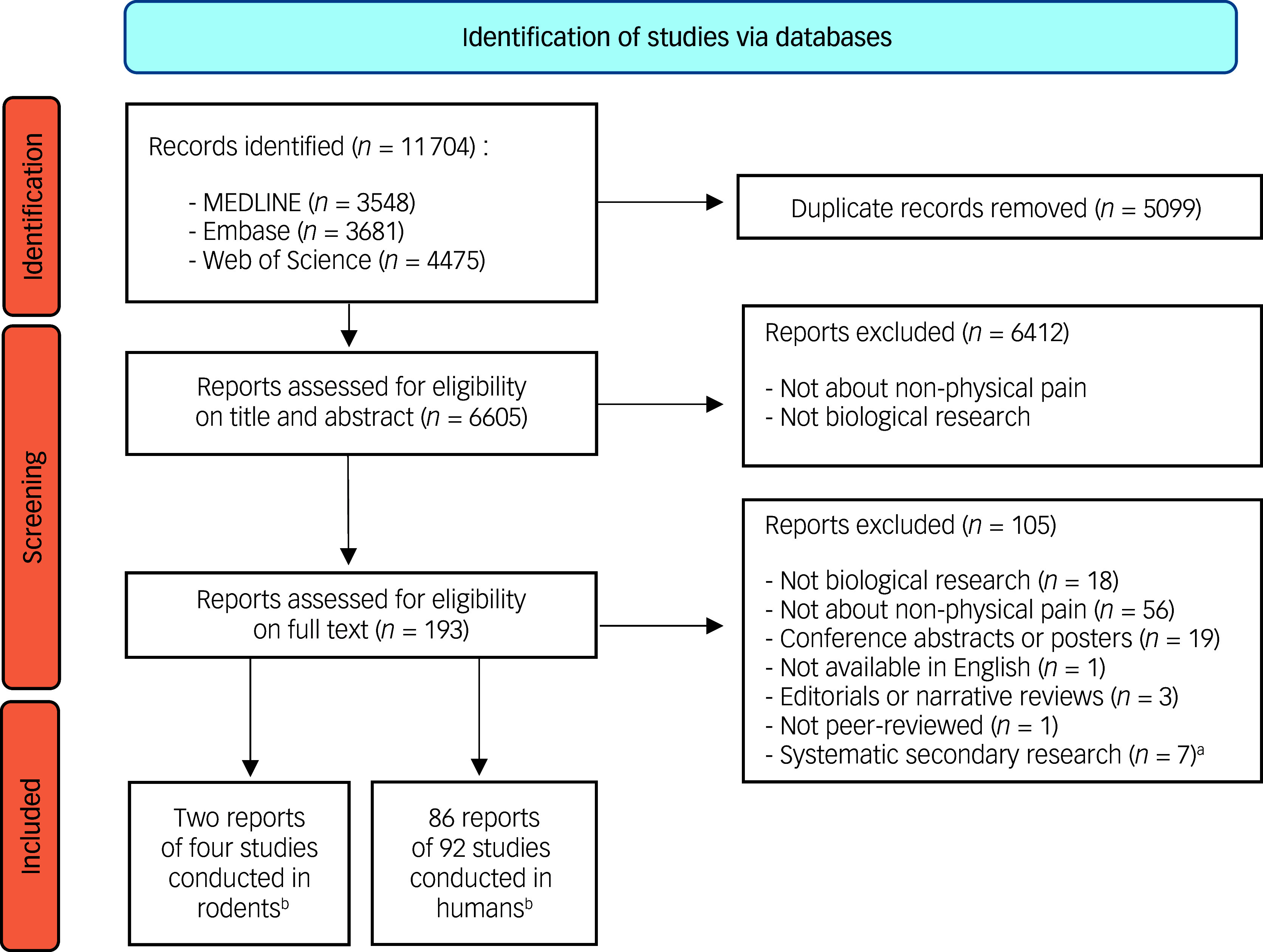
Study flowchart. a. References of systematic secondary research publications were screened in search of additional references of primary research publications that could have been overlooked by the search strategy within the 3 databases. b. A single report could discuss more than one study.

The extraction on 6 November 2023 retrieved 6605 records after removal of duplicates, with 193 full texts searched, resulting in the final inclusion of 88 published reports of primary research studies published between 2003 and 2023 (Fig. 1 and Supplementary material 2). We additionally screened the references of four meta-analyses and three systematic reviews to identify potential additional studies (Supplementary material 3). This screening did not identify any supplemental studies to be included in our screening process. The 88 included reports covered 96 distinct studies, with 4 studies (2 articles) involving rodents and 92 studies (86 articles) involving humans.

### Studies investigating non-physical pain in animal studies

Two articles reported on four studies performed with wild-type rats. All explored the shared biological basis of non-physical pain with physical pain, focusing on the involvement of the endocannabinoid system and on the effect of morphine treatment in their experimental model.^53,54^ To induce non-physical pain, one article reported on a social rejection paradigm and measured non-physical pain as an acute aversive emotional reaction via changes in social interactions (attack behaviour, abnormal vocalisations).^53^ The other used non-social aversive feedback (reward downshift with sucrose) and measured non-physical pain by a drop in sucrose consumption.^54^ Regarding the terms and definitions of non-physical pain used in these studies, one article used ‘social pain’ and ‘social distress’ without providing a clear definition.^53^ The other used the term ‘psychological pain’ and defined it as ‘a negative emotion triggered by reward loss’, following Papini et al.^54,55^

### Studies investigating non-physical pain in humans

Among the 92 human studies, 73.9% (68/92) were experimental (induced non-physical pain in people) and the remainder were observational (26.1%, 24/92), of which 87.5% (21/24) had a cross-sectional design (Table 1). Notably, 27.2% (25/92) of the studies aimed to investigate shared biological bases of non-physical pain with physical pain, corresponding to 20.6% (14/68) of the experimental studies and 45.8% (11/24) of the observational studies.

**Table 1:** Characteristics of the human studies (*n* = 92)

Characteristics	Experimental studies (*n* = 68)	Observational studies (*n* = 24)	All studies^a^ (*n* = 92)
Population setting by continent, *n* (%)			
Asia	19 (27.9)	5 (20.8)	24 (26.1)
Australia	1 (1.5)	1 (4.2)	2 (2.2)
Europe	23 (33.8)	3 (12.5)	26 (28.3)
North America	25 (36.8)	14 (58.3)	39 (42.4)
South America	0 (0.0)	1 (4.2)	1 (1.1)
Total sample size, mean (s.d., min, max)	71 (10, 110, 762)	100 (20, 88, 420)	78 (10, 105, 762)
Health status of participants, *n* (%)			
Healthy	57 (83.8)	9 (37.5)	66 (71.7)
With documented mental disorder^b^	9 (13.2)	15 (62.5)	24 (26.1)
With documented medical condition other than mental disorder^b^	2 (2.9)	0 (0.0)	2 (2.2)
Type of health disorder, if present, *n* (%)^c^	*n* = 11	*n* = 15	*n* = 26
Mood disorders	8 (72.7)	13 (86.7)	21 (80.8)
Suicidal behaviour	6 (54.5)	8 (53.3)	14 (53.8)
Schizophrenia/cocaine use disorder	0 (0.0)	1 (6.7)	1 (3.8)
Complicated grief	1 (9.1)	0 (0.0)	1 (3.8)
Induced termination of pregnancy because of fetal malformation/refractory epilepsy	1 (9.1)	0 (0.0)	1 (3.8)

a. A single report could discuss more than one study, and different studies might have been conducted in the same sample. The 86 reports with human participants covered 92 studies with human participants.

b. Some studies included both healthy and not healthy participants.

c. Some studies included participants with several health disorders.

Overall, the 92 human studies investigated 297 unique biomarkers classified into 14 overarching categories and one ‘Other’ category (Supplementary material 4). The three most investigated overarching categories of biomarker were the prefrontal cortex (44/92, 47.8%), the cingulate cortex (37/92, 40.2%) and the insula (31/92, 33.7%) (Supplementary material 5, Table A). At the biomarker level, the most investigated were the dorsal anterior cingulate cortex (24/92, 26.1%), the anterior insula (18/92, 19.6%), and the ventrolateral prefrontal cortex (16/92, 17.4%) (Supplementary Material 5, Table B). These biomarkers were explored using 28 unique investigation methods, of which fMRI was the most frequently used (38/92, 41.3%), followed by EEG (17/92, 18.5%) and protein expression and kinetic assays (10/92, 10.9%) (Supplementary materials 6 and 7). Concerning the 38 studies utilising fMRI, 71.1% (27/38) used whole brain analysis, 76.3% (29/38) used analyses on specified regions of interest (ROIs) and 28.9% (11/38) used functional connectivity analysis. Of the 11 utilising functional connectivity analysis, 27.3% (3/11) used connectivity graphs.

Overall, the studies encompass 7778 participants and had an average sample size of 78 (s.d. = 105), varying from a minimum of 10 to a maximum of 762 participants. The experimental studies mostly involved healthy individuals (83.8%, 57/68), whereas the observational studies involved more people with mental disorders (62.5%, 15/24). In fact, these 15 observational studies considered mental disorders as an exposure to non-physical pain, whereas the other 9 used the lockdown during the COVID-19 pandemic, loneliness or social exclusion as the exposure. Finally, 25% (6/24) of the observational studies framed non-physical pain as a common experience of human life and involved participants from the general population.

**Fig. 2:**
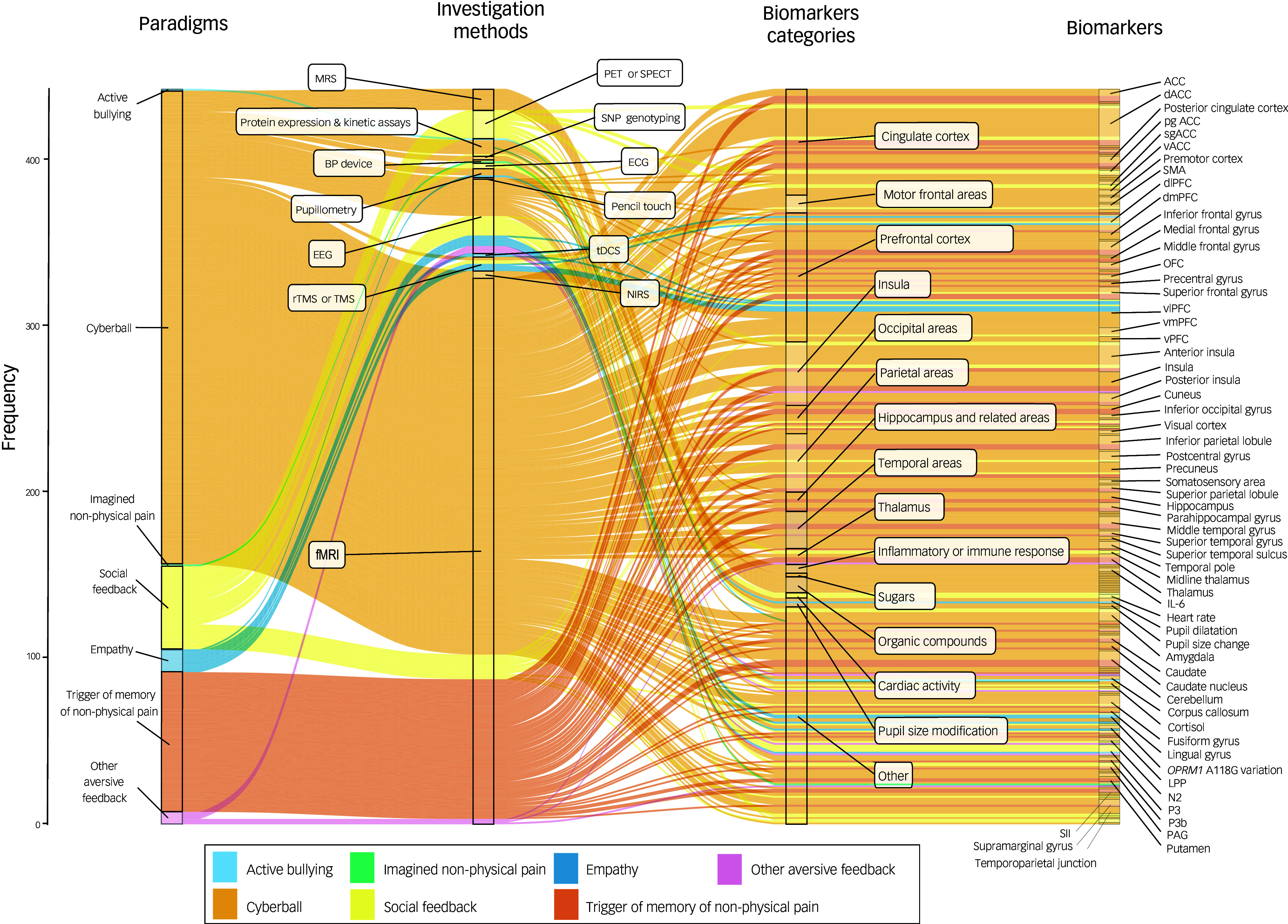
Methodological patterns of investigation in experimental studies of non-physical pain. The Sankey diagram displays the 416 methodological patterns identified across the 68 human experimental studies. Each line represents a methodological pattern, i.e. a combination of an experimental paradigm (first column), an investigation method (second column) and a biomarker (third column for the category and fourth column for the biomarker). Widths of the lines are proportional to the frequency of use of the methodological patterns across the studies. For instance, the use of the cyberball paradigm to investigate the prefrontal cortex using fMRI was the most frequently used methodological pattern (36/416, 8.7%). More precisely, the dlPFC was investigated by 8.33% (3/36) of the investigations of the prefrontal cortex using the cyberball as an experimental paradigm and fMRI as an investigation method. ACC, anterior cingulate cortex; BP, blood pressure; dACC, dorsal ACC; dlPFC, dorsolateral PFC; dmPFC, dorsomedial PFC; ECG, electrocardiogram; EEG, electroencephalogram; fMRI, functional magnetic resonance imaging; IL-6, interleukin 6; LPP, late positive potential; MRS, magnetic resonance spectroscopy; N2, P3 and P3b, event-related potential components; NIRS, near-infrared spectroscopy; OFC, orbitofrontal cortex; PAG, periacqueductal gray; PET/SPECT, positron emission tomography and/or single-photon emission computed tomography; PFC, prefrontal cortex; pgACC, pregenual ACC; rTMS/TMS, repetitive high frequency transcranial magnetic stimulation and/or transcranial magnetic stimulation; sgACC, subgenual ACC; SII, secondary somatosensory cortex; SMA, supplementary motor area; SNP, single nucleotide polymorphism; tDCS, transcranial direct current stimulation; SMA, supplementary motor area; vACC, ventral ACC; vlPFC, ventrolateral PFC; vmPFC, ventromedial PFC; vPFC, ventral PFC.

Table 2 displays the 7 different types of experimental paradigm used to induce non-physical pain across the 68 experimental studies. All had unknown external validity, i.e. it was neither established nor discussed to what extent the non-physical pain deemed to be induced by the paradigm corresponded to non-physical pain observed in clinical populations. The most used paradigm category was the cyberball paradigm (40/68, 58.8%), followed by negative social judgement tasks (12/68, 17.6%) and by paradigms triggering memories of non-physical pain (6/68, 8.8%). Across the 68 experimental studies, 32.4% (22/68) did not report any control for potential confounders in the investigation of the association of the biomarker with non-physical pain. Finally, 17.6 % (12/68) did not report the duration of the induction of non-physical pain. Figure 2 represents in the form of an alluvial plot the 46 unique methodological patterns, i.e. combinations of an experimental model of non-physical pain plus a method of investigation plus a category of biomarker. The most frequent patterns were ‘cyberball–fMRI–cingulate cortex’ and ‘cyberball–fMRI–prefrontal cortex’ (19/68, 27.9%).

**Table 2:** The seven experimental paradigms used across the 68 human experimental studies and their external validity in clinical populations^a^

Experimental paradigm category and its frequency of use across the 68 studies (*n*, %)	Example from studies	External validity in clinical population
Cyberball and variations (40, 58.8%) Non-physical pain is triggered by a social exclusion paradigm where the participant is unexpectedly excluded from a ball-tossing game	Fitzgibbon et al, 2017^56^: ‘a simple online ball tossing game, with two other players where they would take turns to throw the ball to each other (however, this is controlled by a computer program). This task involves two conditions: “inclusion” where the participant is involved in the game of catch and “exclusion” where the participant is initially involved, but shortly becomes left out.’	Unknown
Negative social judgement (12, 7.6%) Non-physical pain is triggered by a sham dating mobile app or sham dating website where the participant is rejected by a potential romantic partner	Hsu et al, 2015^57^: ‘subjects were asked to rate online profiles of preferred-sex individuals with whom they would be most interested in forming a close relationship. A few days after profile ratings were obtained, subjects experienced blocks of feedback in which they were not liked (rejection) or liked (acceptance) by their highest-rated profiles.’	Unknown
Trigger of memory of non-physical pain (6, 8.8%) Non-physical pain is triggered when a participant is exposed to a memory triggering non-physical pain (e.g. grief for a loved one)	O’Connor et al, 2008^58^: ‘Each participant provided a photograph of their deceased loved one […] Participants viewed [pictures of deceased loved ones or stranger pictures] through goggles in randomized order.’	Unknown
Empathy with non-physical pain (6, 8.8%) Non-physical pain is triggered by witnessing another participant experiencing social rejection	Mo et al, 2021^59^: ‘images of social exclusion were […] presented for 5s […] at the start of the passive viewing block, participants were instructed as follows: “In this session, please imagine yourself as the person circled in red and rate the intensity of your unpleasant feelings after viewing the picture.”’	Unknown
Other social aversive feedback (2, 2.9%) Non-physical pain is triggered when a participant is exposed to an aversive social stimulus (e.g. angry faces) which does not constitute a negative social judgement	Heckel et al, 2011^60^: ‘Participants viewed […] aversive facial expressions […] Facial stimuli were drawn from a standardized picture set […] selecting the most angry and most fearful expressions (150% intensity) from the models available’	Unknown
Bullying (1, 1.5%) Non-physical pain is triggered when a participant is bullied by another individual	Priem et al, 2010^61^: ‘Dating partners discussed one participant’s core traits or values. To evoke hurt, one partner was recruited as a confederate and that person was coached to be unsupportive and hurtful in one conversation with the participant. Specifically, the confederate was trained to disconfirm one of the participant’s three core traits or values’	Unknown
Imagined non-physical pain (1, 1.5%) Non-physical pain is triggered when a participant is asked to imagine themself in a situation triggering non-physical pain	Inagaki and Gianaros, 2022^62^: ‘Participants completed an abbreviated […] version of the Liebowitz Social Anxiety Scale […] during which participants imagine how “painful” one feels when participating in hypothetical social experience.’	Unknown

a. Each experimental paradigm is presented with a short description and an example from a study retrieved by the systematic review.

Table 3 presents the different instruments used to measure non-physical pain. Of the 92 studies, 9.8% (9/92) did not measure it at all. Only 18% (17/92 – 14 observational studies and three experimental studies) used one or more scales that were purposively developed to measure non-physical pain, such as the Physical and Psychological Pain Visual Analog Scale (PPP-VAS), the Orbach and Mikulincer Mental Pain Scale (OMMPS) and the Three-Dimensional Psychological Pain Scale (TDPPS).^1^ Overall, 23.9% (22/92) of the studies used one or more *ad hoc* measures of non-physical pain, i.e. measures developed by the authors for the purpose of their study. Furthermore, 50% of studies (46/92) used one or more measurement instruments for other constructs, such as social anxiety, depression, loneliness or threats of fundamental needs. For instance, the Need Threat Questionnaire (NTQ) was the most frequently used by experimental study (31/59, 52.5%).^63^ Finally, 54.2% of the experimental studies (32/59) did not report the timeline for the outcome measure of non-physical pain after the mimicking or induction of the pain.

**Table 3:** Measures of non-physical pain in human studies (*n* = 92)

Measures	Experimental studies, *n* (%)	Observational studies, *n* (%)	All studies, *n* (%)
**Reported measures of non-physical pain^a^**	***n* = 68**	***n* = 24**	***n* = 92**
Self-reported measure^a^	59 (86.8)	23 (95.8)	82 (89.1)
No measure of non-physical pain	9 (13.2)	0 (0.0)	9 (9.8)
Observer-reported measure	0 (0.0)	1 (4.2)	1 (1.1)
**Use of self-reported measures^a^**	***n* = 59**	***n* = 23**	***n* = 82**
Measures primarily developed to measure non-physical pain^a^			
*Ad hoc* measure of non-physical pain (i.e. measures developed by the authors for the purpose of experiments)^a^	19 (32.2)	3 (13.0)	22 (26.8)
PPP-VAS	1 (1.7)	5 (21.7)	6 (7.3)
OMMPS^a^	1 (1.7)	4 (17.4)	5 (6.1)
TDPPS	1 (1.7)	4 (17.4)	5 (6.1)
PAS^a^	0 (0.0)	4 (17.4)	4 (4.9)
Measures primarily developed for other constructs to measure non-physical pain^a^			
NTQ^a^	31 (52.5)	0 (0.0)	31 (37.8)
MSR^a^	1 (1.7)	4 (17.4)	5 (6.1)
BFNE^a^	1 (1.7)	4 (17.4)	5 (6.1)
PANAS^a^	3 (5.1)	0 (0.0)	3 (3.7)
LSAS^a^	2 (3.4)	0 (0.0)	2 (2.4)
BDI^a^	1 (1.7)	1 (4.3)	2 (2.4)
STAI/UCLA Loneliness scale/Yearning/Grief opposed to happiness/ASI^a^	1 (1.7)	1 (4.3)	1 (1.2)

ASI, Addiction Severity Index; BDI, Beck Depression Inventory; BFNE, Brief Fear of Negative Evaluation; LSAS, Liebowitz Social Anxiety Scale; MSR, Mehrabian Sensitivity to Rejection scale; NTQ, Need Threat Questionnaire; OMMPS, Orbach and Mikulincer Mental Pain Scale; PANAS, Positive and Negative Affect Scale; PAS, Psychache Scale; PPP-VAS, Physical and Psychological Pain Visual Analogue Scale; STAI, State Trait Anxiety Inventory; TDPPS, Three-Dimensional Psychological Pain Scale.

a. Some studies used multiple measures.

Regarding the terms and definitions of non-physical pain used, the expression ‘social pain’ (61/86, 70.9%) was most common, followed by ‘psychological pain’ (25/86, 29.1%) and ‘social distress’ (23/86, 26.7%) (Supplementary material 8, Table A). Experimental studies tended to use ‘social pain’ (55/64, 85.9%) and ‘social distress’ (22/64, 34.4%) more, whereas observational studies used ‘psychological pain’ (15/21, 71.4%) and ‘mental pain’ (6/21, 28.6%) more. Regarding the consistency of the terms used within a same study, 58.2% (50/86) used 2 or more terms to refer to non-physical pain (Supplementary material 8, Table B). Overall, only 41.9% (36/86) of the publications reported a definition. Table 4 displays the different definitions of non-physical pain retrieved across these 36 articles. Despite heterogeneity, all definitions agreed on the core feature of non-physical pain being a negative subjective emotional state.

**Table 4:** Terms and definitions of non-physical pain retrieved in human studies (*n* = 86)

Terms (*n*, %)^a^	Definitions	Associated theoretical references
Social pain (22, 66.1%)	Thematic content of the 22 definitions of social pain: Negative emotion caused by an objective social rejection/devaluation, involving same brain regions as physical pain Negative emotion caused by the subjective perception of being socially rejected/devaluated	Eisenberger and Lieberman, 2004: ‘the pain experienced upon social injury when social relationships are threatened, damaged or lost’^64^ Eisenberger, 2012: ‘unpleasant experience that is associated with actual or potential damage to one’s sense of social connection or social value (owing to social rejection, exclusion, negative social evaluation or loss)’^2^ MacDonald and Leary, 2005: ‘a specific emotional reaction to the perception that one is being excluded from desired relationships or being devalued by desired relationship partners or groups’^64^
Psychological pain (12, 33.3%)	Thematic content of the 12 definitions of psychological pain: Unbearable subjective emotional state caused by unmet core needs, potentially leading to suicidality Introspective experience of negative emotions Negative subjective emotional state caused by a perception of the self as being deficient	Shneidman, 1993: ‘the introspective experience of negative emotions such as dread, despair, fear, grief, guilt, frustrated love, loneliness, and loss’^6^ Meerwijk and Weiss, 2011: ‘a lasting, unsustainable, and unpleasant feeling resulting from negative appraisal of an inability or deficiency of the self’^4^
Mental pain (1, 2.8%)	Definition of mental pain retrieved from Reisch et al, 2010:^65^ ‘Mental pain […] defined as subjectively unbearable states of mind related to the suicide attempt’	Orbach et al, 2003: ‘a perception of negative changes in the self and its function that is accompanied by strong negative feeling’^66^
Spiritual pain (1, 2.8%)	Definition of spiritual pain retrieved from Boss et al, 2015:^67^ ‘Despite slightly different definitions, scientists generally agree that spiritual pain is a state of disorder and occurs when a person experiences a conflict, either acute or chronic, between their spiritual beliefs and actual life events’	O’Neil and Mako, 2011: ‘a disruption in the principle which pervades a person’s entire being and which integrates and transcends one’s biological nature’^48^

a. The 86 human publications used 11 terms to refer to non-physical pain, of which only 4 received a definition (social pain, psychological pain, mental pain and spiritual pain) across 36 studies. For mental pain and spiritual pain, we report the only definition retrieved. For social pain and psychological pain, which had several definitions, we synthesised their thematic content based on the qualitative content analysis. The comprehensive results of the qualitative content analysis are reported in Supplementary material 8 Table C.

## Discussion

This methodological systematic review proposed a comprehensive research mapping of the field of studies investigating biological features of non-physical pain. It identified 92 human studies and an additional sample of 4 studies in rodents published in the two past decades. Human studies investigated a total of 297 candidate biomarkers with a repeated pattern of investigation ‘social pain–cyberball–fMRI–cingulate cortex and/or prefrontal cortex’.

We identified 9 conceptual and methodological challenges to improving research on the biological basis of non-physical pain, for which we propose 11 ways forward (See Table A1 under Appendix). Four of these challenges are explored here.

### Conceptual weakness of existing definitions

A first concern regards the conceptual weakness of the field. The distinctions between various terms related to non-physical pain are not clearly defined at the level of individual studies. In fact, 58.2% of the studies use two or more different terms without clarifying how they are similar or different. Additionally, when looking at the sample of studies as a whole, even a single term can be defined in different ways across studies. Additionally, 58.1% of the human studies did not clearly define non-physical pain. The fact that publications use multiple terms with unclarified meaning or that the same term can have several definitions contributes to conceptual confusion. Besides the resulting fragmentation of the research field, this also compromises the validity and interpretability of results, as shown for other concepts such as empathy.^68,69^ Although all definitions retrieved by our study agreed on one core feature of non-physical pain – a negative subjective emotional state – we do not conclude that it should be an operational definition of non-physical pain. Rather, further research that would aim at developing an operational definition would need to at least complement the findings of our study with (a) clinical investigations of non-physical pain (e.g. transcultural studies exploring how people use the word pain or phenomenological descriptions of the lived experience) and (b) examination of other research fields (such as clinical psychology or philosophy).^21,70,71^

### Lack of valid measurement instruments

A second concern regards the measurement of non-physical pain. Most of the studies measured non-physical pain using a tool designed to measure constructs other than non-physical pain (e.g. social anxiety, depression, loneliness). For example, the most frequently used measurement instrument was the Need Threat Questionnaire (NTQ), a scale measuring threatened fundamental needs rather than non-physical pain.^7,20,63,64^ Only 15 studies used a patient-reported outcome developed to measure the construct of non-physical pain. It is worth reminding that, in our previous systematic review, the 10 tools used were assessed to have uncertain content validity using the COSMIN framework.^1^ Just as problematic, the second most frequently used measures were developed *ad hoc*, with little to no reporting of their development or the validation of their psychometric properties.^72^ Additionally, the use of measures tailored for an experimental paradigm questions the possibility of translating the results to non-physical pain observed in clinical populations or even in the general population outside of the context of the paradigm. This is also the case for the NTQ, tailored by Williams et al for social psychology experiments based on cyber-ostracism paradigms (foremost the cyberball), and of course for all other *ad hoc* measures. A final problem with measurement was that 10% of the human studies did not assess whether non-physical pain was present in participants after experimental induction.

### Experimental paradigms and their translationality

A third concern regards the experimental paradigms used to induce non-physical pain. The cyberball paradigm was by far the most commonly used. However, several methodological considerations have been raised regarding the extrapolation of the state induced.^73,74^ It remains unclear whether the pain experienced by people during the cyberball paradigm, or in the other experimental paradigms, is like the non-physical pain experienced either in the context of mental disorders (e.g. non-physical pain in depression) or in non-pathological contexts (e.g. grief, romantic rejection). The repeated use of the ‘social pain–cyberball–fMRI–NTQ’ conceptual and methodological framework tends to forge ‘social pain’ as the state induced by the cyberball experiment. One may speculate that frequent use of the cyberball paradigm may stem from the consistency of its effects on the activity of some brain regions. However, such selection of experimental tasks may lead to a critical lack of reliability of task-based measures at the individual level, a drawback referred to as a ‘reliability paradox’.^75^ Put simply, this paradox might have thrived because seeking for replicable task effects results in selecting tasks with low inter-individual variability, which entails poor ability to capture inter-individual differences. Although the studies retrieved in our review investigated acute non-physical pain triggered by a short exposure to social rejection, there is a need to further explore forms of non-physical pain, in particular chronic forms that might be closer to those experienced by people with mental disorders or triggered by other events.^5,76–78^ This certainly needs the development of new paradigms and measures derived from refined clinical characterisation of non-physical pain.

### Questionable animal models of non-physical pain

A fourth concern regards animal models used in studies to dissect pathophysiological mechanisms involved in non-physical pain. Acknowledging that animals could have equivalent experience, it is challenging to develop a convincing animal model of non-physical pain, as it is for many other psychological constructs derived from human experience (e.g. depression, anhedonia, suicidality). As an echo of the research in humans marked by the cyberball paradigm, of the four studies identified in animals, two used a social defeat paradigm in rodents.^53^ In these studies, non-physical pain is extrapolated from experiences of acute stress caused by a short experience of social defeat. However, the model of social defeat was initially developed to enable an animal model of depression characterised by marked negative and sustainable emotional biases.^79^ Of note, behaviours characterised with the social defeat paradigm can be referred to as hopelessness in animal research, but rarely with the common terms used to refer to non-physical pain, which may explain the paucity of animal studies collected in our search.^79^ Moreover, this paradigm was meant to chronically expose the subjects to social defeat rather than to a short exposure and therefore we might need a better characterisation of the emotional states induced by an acute and a chronic exposure to social defeat (difference and similarities) to link it to non-physical pain in human. This echoes the general concerns about the robustness of research on psychiatric biomarkers and the necessary caution regarding the translational aspect of the behaviours and dimensions assessed.^80–84^

### The reproducibility crisis

A final concern regards the incomplete reporting of methods and methodological features, which are known factors of the ‘reproducibility crisis’.^85,86^ Previous meta-research initiatives have tackled the issues of improving research practices in life sciences, with the development of new guidelines for the reporting, designs and evaluation of animal studies, as well as their evidence synthesis.^87,88^

### Limitations

Our study has several limitations. First, regarding our search strategy, we included 15 terms referring to the state of non-physical pain but, contrary to other studies, we did not include terms referring to situations or paradigms triggering non-physical pain (e.g. grief, social rejection, social exclusion), as these two terms referred to experimental paradigms or objective facts, and not to the subjective experience of non-physical pain.^17,89^ To our knowledge, there are no scientific guidelines for the mapping of conceptual and methodological features across studies. To compensate, we provide an extensive description of our mapping procedure in the interests of reproducibility of our analysis of conceptual features and methodological characteristics.

### Future research

The biomedical literature investigates pains that are not primarily felt in the body, based on the search of shared biological features with physical pain. This research field is hampered by a weak conceptualisation and lack of measurement. Additionally, the use of experimental paradigms to induce non-physical pain and measures with unknown external validity leads to results that cannot be translated in clinical practice. Acknowledging that it is critical to have biological investigation of the breadth of human suffering, this methodological systematic review proposed ways forwards to improve research. Overall, the priority seems to be the development of an agreed international definition and measure of non-physical pain to be used both in observational and experimental research and in clinical practice to improve the consistency and translationality of the results. The foundation of a scientific terminology is to use unambiguous, stable terms that are universally adopted, even if this comes at the cost of some reductionism. It is worth mentioning that, as semantic heterogeneity occurs frequently in psychopathological concepts, attempts to harmonise the terminology relating to non-physical pain would inadvertently rely on auxiliary psychological notions which in turn will require operational clarification. Hence, a certain degree of circularity will probably have to be tolerated for any potential definition of non-physical pain. Nevertheless, concerning for instance physical pain, the agreement on a definition of physical pain in 1976 by the International Association for the Study of Pain accelerated both research and the development of therapeutic interventions.^90^

## Supporting information

Duranté et al. supplementary material Duranté et al. supplementary material

## Data Availability

Qualitative and quantitative extracted data will be shared on reasonable request to the corresponding author.

## Supplementary material

The supplementary material is available online at https://doi.org/10.1192/bjp.2024.292.

## Appendix

**Table A1:** Challenges and ways to improve research on the biological basis of non-physical pain

Challenges of investigations of the biological basis of non-physical pain	Ways forward to improve the quality of research
**Terms and definitions**	
Heterogeneity of terms used to mean a ‘pain not primarily felt in the body’ Heterogeneity of definitions Conceptual weakness of the existing definitions (tautological definitions e.g. ‘pain is a painful feeling’, or definitions by examples)	Harmonise terminology Develop a definition by fostering collaborative and interdisciplinary consensus
**Study design**	
Repeated investigation of a subgroup of candidate biomarkers of non-physical pain with the same methodological patterns	Coordinate research effort to limit redundancies and gaps while fostering reproducibility^86,91–94^
Lack of prospective observational studies allowing to examine whether the biomarker associated with non-physical pain correlates with changes in non-physical pain over time	Implement prospective observational studies with repeated measure
**Experimental paradigms**	
Unknown external validity of animal models of non-physical pain	Foster the developement of animal models of psychological constructs that are relevant to human experiences^(79,80,95)^
Unknown external validity of experimental paradigms regarding the experience of non-physical pain in clinical and general populations	Ensure that the non-physical pain induced by the experience is alike non-physical pain felt in normal situation (e.g. grief) or pathological (e.g. depression) Foster interdisciplinary and translational collaborations^95–97^ Involve people with lived-experience, to prepare for a better translationality of the results^98,99^
Rare consideration of confounders in the experimental paradigm which limits causal inference	Develop experimental paradigms disentangling non-physical pain from confounding mental states (e.g. surprise)
Incomplete description of the experimental paradigms (e.g. duration of the induction of non-physical pain)	Foster reporting guidelines conceived for psychological and experimental research^100–102^
**Measurement instruments**	
Lack of measure of non-physical pain, or use of unspecific measure, or use of debatable measures to check whether non-physical pain was induced in the subjects	Use a measure of non-physical pain validated in the population of interest, and develop a measure if none is adequate^1,94^
