# Supplementary material for: Biological research on mental pain, social pain and other pains not primarily felt in the body: methodological systematic review
Source: Br J Psychiatry. 2025 Mar 21;227(4):707–17. doi: 10.1192/bjp.2024.292 (PMC12492066; doi:10.1192/bjp.2024.292)
Supplement: Duranté et al. supplementary material [file S0007125024002927sup001.docx]

**Supplementary material for the manuscript by Duranté *et al.* 2024**

[Supplementary material 1: Search strings performed on the 3 databases on November 6^h^, 2023 2](#_Toc169701755)

[Supplementary material 2: Publications of primary research studies included in the systematic review 3](#_Toc169701756)

[Supplementary material 3: List of the 7 meta-analyses and systematic reviews screened for additional references 10](#_Toc169701757)

[Supplementary material 4: Categories of biomarkers investigated by human studies 11](#_Toc169701758)

[Supplementary material 5: Biomarkers investigated by human studies 12](#_Toc169701759)

[Supplementary material 6: Categories of methods of investigation of the biomarkers used in human studies 14](#_Toc169701760)

[Supplementary material 7: Methods of investigation of the biomarkers 15](#_Toc169701761)

[Supplementary material 8: Terms and definitions of non-physical pain used in human publications 16](#_Toc169701762)

# Supplementary material 1: Search strings performed on the 3 databases on November 6^h^, 2023

MEDLINE via PubMed:

("mental pain"[TW]) OR ("psychic pain" [TW]) OR ("psychache*"[TW]) OR ("psychological pain"[TW]) OR ("spiritual pain"[TW]) OR ("social pain"[TW]) OR ("emotional pain"[TW]) OR ("soul pain"[TW]) OR ("psychalg*"[TW]) OR ("painful feeling*"[TW]) OR ("hurt feeling*"[TW]) OR ("mental suffer*" [TW]) OR ("emotional suffer*" [TW]) OR ("psychological suffer*" [TW]) OR ("social distress" [TW])

EMBASE via embase.com:

('mental pain':ti,ab,kw OR 'psychalg*':ti,ab,kw OR 'psychache*':ti,ab,kw OR 'psychic pain':ti,ab,kw OR 'soul pain':ti,ab,kw OR 'painful feeling*':ti,ab,kw OR 'psychological pain':ti,ab,kw OR 'spiritual pain':ti,ab,kw OR 'social pain':ti,ab,kw OR 'emotional pain':ti,ab,kw OR 'hurt feeling*':ti,ab,kw OR 'psychological suffer*':ti,ab,kw OR 'mental suffer*':ti,ab,kw OR 'emotional suffer*':ti,ab,kw OR 'social distress':ti,ab,kw) AND [embase]/lim

Web Of Science via Clarivate:

TS=("mental pain") OR TS=("psychological pain") OR TS=("psychic pain") OR TS=("psychache*") OR TS=("social pain") OR TS=("emotional pain")OR TS=("spiritual pain") OR TS=("soul pain") OR TS=("psychalg*") OR TS=("painful feeling*") OR TS=("hurt feeling*") OR TS=("psychological suffer*") OR TS=("emotional suffer*") OR TS=("mental suffer*") OR TS=("social distress")

# Supplementary material 2: Publications of primary research studies included in the systematic review

1. Asscheman, J. Susanne, Susanne Koot, Ili Ma, J. Marieke Buil, Lydia Krabbendam, Antonius H.N. Cillessen, and Pol A.C. Van Lier. “Heightened Neural Sensitivity to Social Exclusion in Boys with a History of Low Peer Preference during Primary School.” *Developmental Cognitive Neuroscience* 38 (August 2019): 100673. <https://doi.org/10.1016/j.dcn.2019.100673>.
2. Bass, Ellyn Charlotte, Sarah Josephine Stednitz, Kevin Simonson, Tori Shen, and Ethan Gahtan. “Physiological Stress Reactivity and Empathy Following Social Exclusion: A Test of the Defensive Emotional Analgesia Hypothesis.” *Social Neuroscience* 9, no. 5 (September 3, 2014): 504–13. <https://doi.org/10.1080/17470919.2014.929533>.
3. Bohaterewicz, Bartosz, Anna Maria Sobczak, Alicja Krześniak, Dagmara Mętel, and Przemysław Adamczyk. “On the Relation of Gyrification and Cortical Thickness Alterations to the Suicidal Risk and Mental Pain in Chronic Schizophrenia Outpatients.” *Psychiatry Research: Neuroimaging* 316 (October 2021): 111343. <https://doi.org/10.1016/j.pscychresns.2021.111343>.
4. Bonenberger, M., P. L. Plener, R. C. Groschwitz, G. Grön, and B. Abler. “Polymorphism in the Μ-Opioid Receptor Gene (OPRM1) Modulates Neural Processing of Physical Pain, Social Rejection and Error Processing.” *Experimental Brain Research* 233, no. 9 (September 2015): 2517–26. <https://doi.org/10.1007/s00221-015-4322-9>.
5. Borelli, Eleonora, Francesca Benuzzi, Daniela Ballotta, Elena Bandieri, Mario Luppi, Cristina Cacciari, Carlo Adolfo Porro, and Fausta Lui. “Words Hurt: Common and Distinct Neural Substrates Underlying Nociceptive and Semantic Pain.” *Frontiers in Neuroscience* 17 (September 27, 2023): 1234286. <https://doi.org/10.3389/fnins.2023.1234286>.
6. Boss, Lisa, Sandy Branson, Stanley Cron, and Duck-Hee Kang. “Spiritual Pain in Meals on Wheels’ Clients.” *Healthcare* 3, no. 4 (October 10, 2015): 917–32. <https://doi.org/10.3390/healthcare3040917>.
7. Cáceda, Ricardo, W Sue T Griffin, and Pedro L Delgado. “A Probe in the Connection between Inflammation, Cognition and Suicide.” *Journal of Psychopharmacology* 32, no. 4 (April 2018): 482–88. <https://doi.org/10.1177/0269881118764022>.
8. Cáceda, Ricardo, G. Andrew James, Zachary N. Stowe, Pedro L. Delgado, Nolan Kordsmeier, and Clint D. Kilts. “The Neural Correlates of Low Social Integration as a Risk Factor for Suicide.” *European Archives of Psychiatry and Clinical Neuroscience* 270, no. 5 (August 2020): 619–31. <https://doi.org/10.1007/s00406-019-00990-6>.
9. Cao, Yunfei, Qing Wei, Shiquan Gui, and Fuhong Li. “The Temporal Course of Vicarious Embarrassment: An Electrophysiological Study.” *Social Neuroscience* 15, no. 4 (July 3, 2020): 435–46. <https://doi.org/10.1080/17470919.2020.1754288>.
10. Cascio, Christopher N., Sara H. Konrath, and Emily B. Falk. “Narcissists’ Social Pain Seen Only in the Brain.” *Social Cognitive and Affective Neuroscience* 10, no. 3 (March 1, 2015): 335–41. <https://doi.org/10.1093/scan/nsu072>.
11. Conejero, I, I. Jaussent, A. Cazals, E. Thouvenot, T. Mura, E. Le Bars, S. Guillaume, S. Squalli, P. Courtet, and E. Olié. “Association between Baseline Pro-Inflammatory Cytokines and Brain Activation during Social Exclusion in Patients with Vulnerability to Suicide and Depressive Disorder.” *Psychoneuroendocrinology* 99 (January 2019): 236–42. <https://doi.org/10.1016/j.psyneuen.2018.09.041>.
12. Cristofori, Irene, Laura Moretti, Sylvain Harquel, Andres Posada, Gianluca Deiana, Jean Isnard, François Mauguière, and Angela Sirigu. “Theta Signal as the Neural Signature of Social Exclusion.” *Cerebral Cortex* 23, no. 10 (October 2013): 2437–47. <https://doi.org/10.1093/cercor/bhs236>.
13. Dalgleish, Tim, Nicholas D. Walsh, Dean Mobbs, Susanne Schweizer, Anne-Laura Van Harmelen, Barnaby Dunn, Valerie Dunn, Ian Goodyer, and Jason Stretton. “Social Pain and Social Gain in the Adolescent Brain: A Common Neural Circuitry Underlying Both Positive and Negative Social Evaluation.” *Scientific Reports* 7, no. 1 (February 7, 2017): 42010. <https://doi.org/10.1038/srep42010>.
14. DeWall, C. Nathan, Geoff MacDonald, Gregory D. Webster, Carrie L. Masten, Roy F. Baumeister, Caitlin Powell, David Combs, et al. “Acetaminophen Reduces Social Pain: Behavioral and Neural Evidence.” *Psychological Science* 21, no. 7 (July 2010): 931–37. <https://doi.org/10.1177/0956797610374741>.
15. Eisenberger, Naomi I., Shelly L. Gable, and Matthew D. Lieberman. “Functional Magnetic Resonance Imaging Responses Relate to Differences in Real-World Social Experience.” *Emotion* 7, no. 4 (2007): 745–54. <https://doi.org/10.1037/1528-3542.7.4.745>.
16. Eisenberger, Naomi I., Tristen K. Inagaki, Lian T. Rameson, Nehjla M. Mashal, and Michael R. Irwin. “An fMRI Study of Cytokine-Induced Depressed Mood and Social Pain: The Role of Sex Differences.” *NeuroImage* 47, no. 3 (September 2009): 881–90. <https://doi.org/10.1016/j.neuroimage.2009.04.040>.
17. Eisenberger, Naomi I., Matthew D. Lieberman, and Kipling D. Williams. “Does Rejection Hurt? An fMRI Study of Social Exclusion.” *Science* 302, no. 5643 (October 10, 2003): 290–92. <https://doi.org/10.1126/science.1089134>.
18. Fang, Shulin, Samuel F. Law, Xinlei Ji, Qinyu Liu, Panwen Zhang, Runqing Zhong, Huanhuan Li, Xiaosheng Wang, Shuqiao Yao, and Xiang Wang. “Potential Neuropsychological Mechanism Involved in the Transition from Suicide Ideation to Action – a Resting-State fMRI Study Implicating the Insula.” *European Psychiatry* 66, no. 1 (2023): e69. <https://doi.org/10.1192/j.eurpsy.2023.2444>.
19. Fitzgibbon, Bernadette Mary, Melissa Kirkovski, Neil Wayne Bailey, Richard Hilton Thomson, Naomi Eisenberger, Peter Gregory Enticott, and Paul Bernard Fitzgerald. “Low-Frequency Brain Stimulation to the Left Dorsolateral Prefrontal Cortex Increases the Negative Impact of Social Exclusion among Those High in Personal Distress.” *Social Neuroscience* 12, no. 3 (May 4, 2017): 237–41. <https://doi.org/10.1080/17470919.2016.1166154>.
20. Gunther Moor, Bregtje, Eveline A. Crone, and Maurits W. Van Der Molen. “The Heartbrake of Social Rejection: Heart Rate Deceleration in Response to Unexpected Peer Rejection.” *Psychological Science* 21, no. 9 (September 2010): 1326–33. <https://doi.org/10.1177/0956797610379236>.
21. Hao, Ziyu, Huanhuan Li, and Yixuan Lin. “The Characterization of Static and Dynamic Brain Functional Networks in Suicide Attempters with Major Depressive Disorder and Its Relation to Psychological Pain.” *Psychiatry Research: Neuroimaging* 327 (December 2022): 111562. <https://doi.org/10.1016/j.pscychresns.2022.111562>.
22. Hao, Ziyu, Huanhuan Li, Lisheng Ouyang, Fang Sun, Xiaotong Wen, and Xiang Wang. “Pain Avoidance and Functional Connectivity between Insula and Amygdala Identifies Suicidal Attempters in Patients with Major Depressive Disorder Using Machine Learning.” *Psychophysiology* 60, no. 1 (January 2023): e14136. <https://doi.org/10.1111/psyp.14136>.
23. He, Zhenhong, Jun Zhao, Junshi Shen, Nils Muhlert, Rebecca Elliott, and Dandan Zhang. “The Right VLPFC and Downregulation of Social Pain: A TMS Study.” *Human Brain Mapping* 41, no. 5 (April 2020): 1362–71. <https://doi.org/10.1002/hbm.24881>.
24. Heckel, Andreas, Christoph Rothmayr, Katharina Rosengarth, Göran Hajak, Mark W. Greenlee, and Peter Eichhammer. “Aversive Faces Activate Pain Responsive Regions in the Brain.” *NeuroReport* 22, no. 11 (August 3, 2011): 548–53. <https://doi.org/10.1097/WNR.0b013e328348b54a>.
25. Heeringen, Kees van. “The Functional Neuroanatomy of Mental Pain in Depression.” *Psychiatry Research*, 2010, 4. <https://doi.org/10.1016/j.pscychresns.2009.07.011>.
26. Hofman, Simon, Matthias J. Wieser, and Frederik M. Van Der Veen. “Acetaminophen Does Not Affect Cardiac and Brain Responses to Social Rejection but Seems to Attenuate Behavioral Adaptation in a Social Judgment Task.” *Social Neuroscience* 16, no. 4 (July 4, 2021): 362–74. <https://doi.org/10.1080/17470919.2021.1924260>.
27. Hsu, D T, B J Sanford, K K Meyers, T M Love, K E Hazlett, S J Walker, B J Mickey, R A Koeppe, S A Langenecker, and J-K Zubieta. “It Still Hurts: Altered Endogenous Opioid Activity in the Brain during Social Rejection and Acceptance in Major Depressive Disorder.” *Molecular Psychiatry* 20, no. 2 (February 2015): 193–200. <https://doi.org/10.1038/mp.2014.185>.
28. Hsu, D T, B J Sanford, K K Meyers, T M Love, K E Hazlett, H Wang, L Ni, et al. “Response of the μ-Opioid System to Social Rejection and Acceptance.” *Molecular Psychiatry* 18, no. 11 (November 2013): 1211–17. <https://doi.org/10.1038/mp.2013.96>.
29. Hwang, Hyesung G., Jihyun Suh, Jared Balbona, Shreya Sodhi, and Lori Markson. “The Crude Ostracism Detection System: Pupils React to Minimal Cues of Exclusion.” *Journal of Social and Personal Relationships* 37, no. 4 (April 2020): 1225–44. <https://doi.org/10.1177/0265407519891242>.
30. Inagaki, Tristen K., and Peter J. Gianaros. “Resting (Tonic) Blood Pressure Is Associated With Sensitivity to Imagined and Acute Experiences of Social Pain: Evidence From Three Studies.” *Psychological Science* 33, no. 6 (June 2022): 984–98. <https://doi.org/10.1177/09567976211061107>.
31. Inagaki, Tristen K., J. Richard Jennings, Naomi I. Eisenberger, and Peter J. Gianaros. “Taking Rejection to Heart: Associations between Blood Pressure and Sensitivity to Social Pain.” *Biological Psychology* 139 (November 2018): 87–95. <https://doi.org/10.1016/j.biopsycho.2018.10.007>.
32. Jollant, Fabrice, Jamie Near, Gustavo Turecki, and Stéphane Richard-Devantoy. “Spectroscopy Markers of Suicidal Risk and Mental Pain in Depressed Patients.” *Progress in Neuro-Psychopharmacology and Biological Psychiatry* 73 (February 2017): 64–71. <https://doi.org/10.1016/j.pnpbp.2016.10.005>.
33. Jollant, Fabrice, Fabricio Perreira, Laura M. Fiori, Stéphane Richard-Devantoy, Pierre-Eric Lutz, Raoul Belzeaux, and Gustavo Turecki. “Neural and Molecular Correlates of Psychological Pain during Major Depression, and Its Link with Suicidal Ideas.” *Progress in Neuro-Psychopharmacology and Biological Psychiatry* 100 (June 2020): 109909. <https://doi.org/10.1016/j.pnpbp.2020.109909>.
34. Kawamoto, Taishi, Keiichi Onoda, Ken’ichiro Nakashima, Hiroshi Nittono, Shuhei Yamaguchi, and Mitsuhiro Ura. “Is Dorsal Anterior Cingulate Cortex Activation in Response to Social Exclusion Due to Expectancy Violation? An fMRI Study.” *Frontiers in Evolutionary Neuroscience* 4 (2012). <https://doi.org/10.3389/fnevo.2012.00011>.
35. Kersting, Anette, Patricia Ohrmann, Anya Pedersen, Kristin Kroker, Daniela Samberg, Jochen Bauer, Harald Kugel, et al. “Neural Activation Underlying Acute Grief in Women After the Loss of an Unborn Child.” *American Journal of Psychiatry* 166, no. 12 (December 2009): 1402–10. <https://doi.org/10.1176/appi.ajp.2009.08121875>.
36. Kim, Chong-Su, Go-Eun Shin, Yunju Cheong, Ji‑Hee Shin, Dong-Mi Shin, and Woo Young Chun. “Experiencing Social Exclusion Changes Gut Microbiota Composition.” *Translational Psychiatry* 12, no. 1 (June 17, 2022): 254. <https://doi.org/10.1038/s41398-022-02023-8>.
37. Kim, Diane J., Sarah J. Blossom, Pedro L. Delgado, Jessica M. Carbajal, and Ricardo Cáceda. “Examination of Pain Threshold and Neuropeptides in Patients with Acute Suicide Risk.” *Progress in Neuro-Psychopharmacology and Biological Psychiatry* 95 (December 2019): 109705. <https://doi.org/10.1016/j.pnpbp.2019.109705>.
38. Kim, Hairin, Seyul Kwak, Elisa C Baek, Naeun Oh, Ekaterina Baldina, Yoosik Youm, and Jeanyung Chey. “Brain Connectivity during Social Exclusion Differs Depending on the Closeness within a Triad among Older Adults Living in a Village.” *Social Cognitive and Affective Neuroscience* 18, no. 1 (April 27, 2023): nsad015. <https://doi.org/10.1093/scan/nsad015>.
39. Krill, Austen. “In-Group and out-Group Membership Mediates Anterior Cingulate Activation to Social Exclusion.” *Frontiers in Evolutionary Neuroscience* 1 (2009). <https://doi.org/10.3389/neuro.18.001.2009>.
40. Kross, Ethan, Marc G. Berman, Walter Mischel, Edward E. Smith, and Tor D. Wager. “Social Rejection Shares Somatosensory Representations with Physical Pain.” *Proceedings of the National Academy of Sciences* 108, no. 15 (April 12, 2011): 6270–75. <https://doi.org/10.1073/pnas.1102693108>.
41. Lelieveld, Gert-Jan, Bregtje Gunther Moor, Eveline A. Crone, Johan C. Karremans, and Ilja Van Beest. “A Penny for Your Pain? The Financial Compensation of Social Pain After Exclusion.” *Social Psychological and Personality Science* 4, no. 2 (March 2013): 206–14. <https://doi.org/10.1177/1948550612446661>.
42. Li, Sijin, Jingxu Chen, Kexiang Gao, Feng Xu, and Dandan Zhang. “Excitatory Brain Stimulation over the Left Dorsolateral Prefrontal Cortex Enhances Voluntary Distraction in Depressed Patients.” *Psychological Medicine* 53, no. 14 (October 2023): 6646–55. <https://doi.org/10.1017/S0033291723000028>.
43. Li, Sijin, Hui Xie, Zixin Zheng, Weimao Chen, Feng Xu, Xiaoqing Hu, and Dandan Zhang. “The Causal Role of the Bilateral Ventrolateral Prefrontal Cortices on Emotion Regulation of Social Feedback.” *Human Brain Mapping* 43, no. 9 (June 15, 2022): 2898–2910. <https://doi.org/10.1002/hbm.25824>.
44. Martelli, Alexandra M, David S Chester, Kirk Warren Brown, Naomi I Eisenberger, and C Nathan DeWall. “When Less Is More: Mindfulness Predicts Adaptive Affective Responding to Rejection via Reduced Prefrontal Recruitment.” *Social Cognitive and Affective Neuroscience* 13, no. 6 (June 1, 2018): 648–55. <https://doi.org/10.1093/scan/nsy037>.
45. Meerwijk, Esther L., Catherine A. Chesla, and Sandra J. Weiss. “Psychological Pain and Reduced Resting-State Heart Rate Variability in Adults with a History of Depression: Psychological Pain and Resting-State Physiology.” *Psychophysiology* 51, no. 3 (March 2014): 247–56. <https://doi.org/10.1111/psyp.12175>.
46. Meerwijk, Esther L., Judith M. Ford, and Sandra J. Weiss. “Resting-State EEG Delta Power Is Associated with Psychological Pain in Adults with a History of Depression.” *Biological Psychology* 105 (February 2015): 106–14. <https://doi.org/10.1016/j.biopsycho.2015.01.003>.
47. Meerwijk, Esther L., and Sandra J. Weiss. “Does Suicidal Desire Moderate the Association between Frontal Delta Power and Psychological Pain?” *PeerJ* 4 (January 4, 2016): e1538. <https://doi.org/10.7717/peerj.1538>.
48. Minervini, Anthony, Adriana LaVarco, Samantha Zorns, Ruth Propper, Christos Suriano, and Julian Paul Keenan. “Excitatory Dorsal Lateral Prefrontal Cortex Transcranial Magnetic Stimulation Increases Social Anxiety.” *Brain Sciences* 13, no. 7 (June 24, 2023): 989. <https://doi.org/10.3390/brainsci13070989>.
49. Mo, Licheng, Tianyou Guo, Yueyao Zhang, Feng Xu, and Dandan Zhang. “The Role of Ventrolateral Prefrontal Cortex on Emotional Regulation of Social Pain in Depressed Patients: A TMS Study.” *Acta Psychologica Sinica* 53, no. 5 (2021): 494. <https://doi.org/10.3724/SP.J.1041.2021.00494>.
50. Mo, Licheng, Sijin Li, Si Cheng, Yiwei Li, Feng Xu, and Dandan Zhang. “Emotion Regulation of Social Pain: Double Dissociation of Lateral Prefrontal Cortices Supporting Reappraisal and Distraction.” *Social Cognitive and Affective Neuroscience* 18, no. 1 (September 7, 2023): nsad043. <https://doi.org/10.1093/scan/nsad043>.
51. O’Connor, Mary-Frances, David K. Wellisch, Annette L. Stanton, Naomi I. Eisenberger, Michael R. Irwin, and Matthew D. Lieberman. “Craving Love? Enduring Grief Activates Brain’s Reward Center.” *NeuroImage* 42, no. 2 (August 2008): 969–72. <https://doi.org/10.1016/j.neuroimage.2008.04.256>.
52. Olié, Emilie, Mathilde Husky, Emmanuelle Le Bars, Jeremy Deverdun, Nicolas Menjot De Champfleur, Adrian Alacreu Crespo, Joel Swendsen, and Philippe Courtet. “Prefrontal Activity during Experimental Ostracism and Daily Psychache in Suicide Attempters.” *Journal of Affective Disorders* 285 (April 2021): 63–68. <https://doi.org/10.1016/j.jad.2021.01.087>.
53. Olié, Emilie, Fabrice Jollant, Jeremy Deverdun, Nicolas Menjot De Champfleur, Fabienne Cyprien, Emmanuelle Le Bars, Thibaut Mura, Alain Bonafé, and Philippe Courtet. “The Experience of Social Exclusion in Women with a History of Suicidal Acts: A Neuroimaging Study.” *Scientific Reports* 7, no. 1 (March 7, 2017): 89. <https://doi.org/10.1038/s41598-017-00211-x>.
54. Onoda, Keiichi, Yasumasa Okamoto, Ken’ichiro Nakashima, Hiroshi Nittono, Mitsuhiro Ura, and Shigeto Yamawaki. “Decreased Ventral Anterior Cingulate Cortex Activity Is Associated with Reduced Social Pain during Emotional Support.” *Social Neuroscience* 4, no. 5 (October 2009): 443–54. <https://doi.org/10.1080/17470910902955884>.
55. Onoda, Keiichi, Yasumasa Okamoto, Ken’ichiro Nakashima, Hiroshi Nittono, Shinpei Yoshimura, Sigeto Yamawaki, Shuhei Yamaguchi, and Mitsuhiro Ura. “Does Low Self-Esteem Enhance Social Pain? The Relationship between Trait Self-Esteem and Anterior Cingulate Cortex Activation Induced by Ostracism.” *Social Cognitive and Affective Neuroscience* 5, no. 4 (December 1, 2010): 385–91. <https://doi.org/10.1093/scan/nsq002>.
56. Perini, Irene, Per A. Gustafsson, J. Paul Hamilton, Robin Kämpe, Maria Zetterqvist, and Markus Heilig. “The Salience of Self, Not Social Pain, Is Encoded by Dorsal Anterior Cingulate and Insula.” *Scientific Reports* 8, no. 1 (April 18, 2018): 6165. <https://doi.org/10.1038/s41598-018-24658-8>.
57. Persson, Emil, Erkin Asutay, Markus Heilig, Andreas Löfberg, Nancy Pedersen, Daniel Västfjäll, and Gustav Tinghög. “Variation in the μ-Opioid Receptor Gene ( *OPRM1* ) Does Not Moderate Social-Rejection Sensitivity in Humans.” *Psychological Science* 30, no. 7 (July 2019): 1050–62. <https://doi.org/10.1177/0956797619849894>.
58. Preller, Katrin H., Thomas Pokorny, Andreas Hock, Rainer Kraehenmann, Philipp Stämpfli, Erich Seifritz, Milan Scheidegger, and Franz X. Vollenweider. “Effects of Serotonin 2A/1A Receptor Stimulation on Social Exclusion Processing.” *Proceedings of the National Academy of Sciences* 113, no. 18 (May 3, 2016): 5119–24. <https://doi.org/10.1073/pnas.1524187113>.
59. Priem, Jennifer S., Rachel M. McLaren, and Denise Haunani Solomon. “Relational Messages, Perceptions of Hurt, and Biological Stress Reactions to a Disconfirming Interaction.” *Communication Research* 37, no. 1 (February 2010): 48–72. <https://doi.org/10.1177/0093650209351470>.
60. Reisch, Thomas, Erich Seifritz, Fabrizio Esposito, Roland Wiest, Ladislav Valach, and Konrad Michel. “An fMRI Study on Mental Pain and Suicidal Behavior.” *Journal of Affective Disorders* 126, no. 1–2 (October 2010): 321–25. <https://doi.org/10.1016/j.jad.2010.03.005>.
61. Richard-Devantoy, S., Y. Ding, M. Lepage, G. Turecki, and F. Jollant. “Cognitive Inhibition in Depression and Suicidal Behavior: A Neuroimaging Study.” *Psychological Medicine* 46, no. 5 (April 2016): 933–44. <https://doi.org/10.1017/S0033291715002421>.
62. Rudolph, Karen D., Michelle E. Miernicki, Wendy Troop-Gordon, Megan M. Davis, and Eva H. Telzer. “Adding Insult to Injury: Neural Sensitivity to Social Exclusion Is Associated with Internalizing Symptoms in Chronically Peer-Victimized Girls.” *Social Cognitive and Affective Neuroscience* 11, no. 5 (May 1, 2016): 829–42. <https://doi.org/10.1093/scan/nsw021>.
63. Ruiz-Salas, Juan C., L. Gonzalo De La Casa, Carmen Torres, and Mauricio R. Papini. “Psychological Pain and Opioid Receptors: Reward Downshift Is Disrupted When Tested in a Context Signaling Morphine.” *Pharmacology Biochemistry and Behavior* 216 (May 2022): 173386. <https://doi.org/10.1016/j.pbb.2022.173386>.
64. Schmälzle, Ralf, Matthew Brook O’Donnell, Javier O. Garcia, Christopher N. Cascio, Joseph Bayer, Danielle S. Bassett, Jean M. Vettel, and Emily B. Falk. “Brain Connectivity Dynamics during Social Interaction Reflect Social Network Structure.” *Proceedings of the National Academy of Sciences* 114, no. 20 (May 16, 2017): 5153–58. <https://doi.org/10.1073/pnas.1616130114>.
65. Schneider, Ekaterina, Dora Hopf, Monika Eckstein, Dirk Scheele, Corina Aguilar-Raab, Sabine C. Herpertz, Valery Grinevich, and Beate Ditzen. “Stress during the COVID-19 Pandemic Moderates Pain Perception and Momentary Oxytocin Levels.” *Journal of Clinical Medicine* 12, no. 6 (March 16, 2023): 2333. <https://doi.org/10.3390/jcm12062333>.
66. Schneider, Peggy, Monique Pätz, Rainer Spanagel, and Miriam Schneider. “Adolescent Social Rejection Alters Pain Processing in a CB1 Receptor Dependent Manner.” *European Neuropsychopharmacology* 26, no. 7 (July 2016): 1201–12. <https://doi.org/10.1016/j.euroneuro.2016.04.007>.
67. Slavich, George M., Molly A. Tartter, Patricia A. Brennan, and Constance Hammen. “Endogenous Opioid System Influences Depressive Reactions to Socially Painful Targeted Rejection Life Events.” *Psychoneuroendocrinology* 49 (November 2014): 141–49. <https://doi.org/10.1016/j.psyneuen.2014.07.009>.
68. Sleegers, Willem W.A., Travis Proulx, and Ilja Van Beest. “The Social Pain of Cyberball: Decreased Pupillary Reactivity to Exclusion Cues.” *Journal of Experimental Social Psychology* 69 (March 2017): 187–200. <https://doi.org/10.1016/j.jesp.2016.08.004>.
69. Song, Wei, Huanhuan Li, Ting Guo, Songyuan Jiang, and Xiang Wang. “Effect of Affective Reward on Cognitive Event‐related Potentials and Its Relationship with Psychological Pain and Suicide Risk among Patients with Major Depressive Disorder.” *Suicide and Life-Threatening Behavior* 49, no. 5 (October 2019): 1290–1306. <https://doi.org/10.1111/sltb.12524>.
70. Song, Wei, Huanhuan Li, Fang Sun, Ting Guo, Songyuan Jiang, and Xiang Wang. “Pain Avoidance and Its Relation to Neural Response to Punishment Characterizes Suicide Attempters with Major Depression Disorder.” *Psychiatry Research* 294 (December 2020): 113507. <https://doi.org/10.1016/j.psychres.2020.113507>.
71. Souza, Manassés Soares, Breno Sanvicente-Vieira, Aline Zaparte, Talita Baptista, Maria Aparecida Nagai, Flávia Rotea Mangone, Ana Carolina Pavanelli, Thiago Wendt Viola, and Rodrigo Grassi-Oliveira. “Cocaine Use Disorder Effects on Blood Oxytocin Levels and OXTR DNA Methylation.” *Neuroscience Letters* 816 (November 2023): 137506. <https://doi.org/10.1016/j.neulet.2023.137506>.
72. Themanson, Jason R., Stephanie M. Khatcherian, Aaron B. Ball, and Peter J. Rosen. “An Event-Related Examination of Neural Activity during Social Interactions.” *Social Cognitive and Affective Neuroscience* 8, no. 6 (August 1, 2013): 727–33. <https://doi.org/10.1093/scan/nss058>.
73. Umeda, Masataka, Teresa M. Leutze, and Tristen K. Inagaki. “Replication and Extension of the Link between the Cardiovascular System and Sensitivity to Social Pain in Healthy Adults.” *Social Neuroscience* 16, no. 3 (May 4, 2021): 265–76. <https://doi.org/10.1080/17470919.2021.1897672>.
74. Van Der Veen, F.M., A. Burdzina, and S.J.E. Langeslag. “Don’t You Want Me, Baby? Cardiac and Electrocortical Concomitants of Romantic Interest and Rejection.” *Biological Psychology* 146 (September 2019): 107707. <https://doi.org/10.1016/j.biopsycho.2019.05.007>.
75. Von Mohr, Mariana, Louise P. Kirsch, and Aikaterini Fotopoulou. “The Soothing Function of Touch: Affective Touch Reduces Feelings of Social Exclusion.” *Scientific Reports* 7, no. 1 (October 18, 2017): 13516. <https://doi.org/10.1038/s41598-017-13355-7>.
76. Wager, Tor D., Lauren Y. Atlas, Martin A. Lindquist, Mathieu Roy, Choong-Wan Woo, and Ethan Kross. “An fMRI-Based Neurologic Signature of Physical Pain.” *New England Journal of Medicine* 368, no. 15 (April 11, 2013): 1388–97. <https://doi.org/10.1056/NEJMoa1204471>.
77. Wasylyshyn, Nick, Brett Hemenway Falk, Javier O Garcia, Christopher N Cascio, Matthew Brook O’Donnell, C Raymond Bingham, Bruce Simons-Morton, Jean M Vettel, and Emily B Falk. “Global Brain Dynamics during Social Exclusion Predict Subsequent Behavioral Conformity.” *Social Cognitive and Affective Neuroscience* 13, no. 2 (February 1, 2018): 182–91. <https://doi.org/10.1093/scan/nsy007>.
78. Way, Baldwin M., Shelley E. Taylor, and Naomi I. Eisenberger. “Variation in the μ-Opioid Receptor Gene ( *OPRM1* ) Is Associated with Dispositional and Neural Sensitivity to Social Rejection.” *Proceedings of the National Academy of Sciences* 106, no. 35 (September 2009): 15079–84. <https://doi.org/10.1073/pnas.0812612106>.
79. Weik, Ella, Naznin Virji-Babul, Urs Ribary, and Christine Tipper. “A Matter of Perspective: Distinct Brain Mechanisms for Evaluating Positive and Negative Social Feedback about Oneself and Another Person.” *Social Neuroscience* 17, no. 3 (May 4, 2022): 193–208. <https://doi.org/10.1080/17470919.2022.2058081>.
80. Weschke, Sarah, and Michael Niedeggen. “Target and Non-Target Processing during Oddball and Cyberball: A Comparative Event-Related Potential Study.” Edited by Christian Schmahl. *PLOS ONE* 11, no. 4 (April 21, 2016): e0153941. <https://doi.org/10.1371/journal.pone.0153941>.
81. Yanagisawa, Kuniaki, Keita Masui, Kaichiro Furutani, Michio Nomura, Mitsuhiro Ura, and Hiroshi Yoshida. “Does Higher General Trust Serve as a Psychosocial Buffer against Social Pain? An NIRS Study of Social Exclusion.” *Social Neuroscience* 6, no. 2 (March 8, 2011): 190–97. <https://doi.org/10.1080/17470919.2010.506139>.
82. Yanagisawa, Kuniaki, Keita Masui, Kaichiro Furutani, Michio Nomura, Hiroshi Yoshida, and Mitsuhiro Ura. “Family Socioeconomic Status Modulates the Coping-Related Neural Response of Offspring.” *Social Cognitive and Affective Neuroscience* 8, no. 6 (August 1, 2013): 617–22. <https://doi.org/10.1093/scan/nss039>.
83. “Temporal Distance Insulates against Immediate Social Pain: An NIRS Study of Social Exclusion.” *Social Neuroscience* 6, no. 4 (August 2011): 377–87. <https://doi.org/10.1080/17470919.2011.559127>.
84. Yanagisawa, Kuniaki, Keita Masui, Keiichi Onoda, Kaichiro Furutani, Michio Nomura, Hiroshi Yoshida, and Mitsuhiro Ura. “The Effects of the Behavioral Inhibition and Activation Systems on Social Inclusion and Exclusion.” *Journal of Experimental Social Psychology* 47, no. 2 (March 2011): 502–5. <https://doi.org/10.1016/j.jesp.2010.11.014>.
85. Yu, Wenwen, Yiwei Li, Xueying Cao, Licheng Mo, Yuming Chen, and Dandan Zhang. “The Role of Ventrolateral Prefrontal Cortex on Voluntary Emotion Regulation of Social Pain.” *Human Brain Mapping* 44, no. 13 (September 2023): 4710–21. <https://doi.org/10.1002/hbm.26411>.
86. Zhang, Qingqing, Tingting Chen, Shanshan Liu, Xinying Liu, Yifan Zhang, Fengqiong Yu, Gong-Jun Ji, Xiaoming Li, and Chunyan Zhu. “Effects of High-Definition Transcranial Direct Current Stimulation on Implicit Emotion Regulation of Social Pain in Healthy Individuals.” *Journal of Affective Disorders* 338 (October 2023): 74–82. <https://doi.org/10.1016/j.jad.2023.05.075>.
87. Zhang, Xukai, Peng Li, Susannah C.S.A. Otieno, Hong Li, and Paavo H.T. Leppänen. “Oxytocin Reduces Romantic Rejection-Induced Pain in Online Speed-Dating as Revealed by Decreased Frontal-Midline Theta Oscillations.” *Psychoneuroendocrinology* 133 (November 2021): 105411. <https://doi.org/10.1016/j.psyneuen.2021.105411>.
88. Zhao, Jun, Licheng Mo, Rong Bi, Zhenhong He, Yuming Chen, Feng Xu, Hui Xie, and Dandan Zhang. “The VLPFC versus the DLPFC in Downregulating Social Pain Using Reappraisal and Distraction Strategies.” *The Journal of Neuroscience* 41, no. 6 (February 10, 2021): 1331–39. <https://doi.org/10.1523/JNEUROSCI.1906-20.2020>.

# Supplementary material 3: List of the 7 meta-analyses and systematic reviews screened for additional references

We screened the references of 7 meta-analyses and systematic reviews identified by our search strategy. Through this screening, we did not identify supplemental studies to be included in our study.

1. Cacioppo S, Frum C, Asp E, Weiss RM, Lewis JW, Cacioppo JT. A Quantitative Meta-Analysis of Functional Imaging Studies of Social Rejection. Sci Rep. 2013 Jun 19;3(1):2027.
2. Colino L, Herranz-Herrer J, Gil-Benito E, Ponte-Lopez T, Del Sol-Calderon P, Rodrigo-Yanguas M, et al. Cannabinoid Receptors, Mental Pain and Suicidal Behavior: a Systematic Review. Curr Psychiatry Rep. 2018 Mar 15;20(3):19.
3. Ducasse D, Courtet P, Olié E. Physical and Social Pains in Borderline Disorder and Neuroanatomical Correlates: A Systematic Review. Curr Psychiatry Rep. 2014;
4. Meerwijk EL, Ford JM, Weiss SJ. Brain regions associated with psychological pain: implications for a neural network and its relationship to physical pain. Brain Imaging and Behavior. 2013 Mar;7(1):1–14.
5. Mwilambwe-Tshilobo L, Spreng RN. Social exclusion reliably engages the default network: A meta-analysis of Cyberball. Neuroimage. 2021 Feb 15;227:117666.
6. Rotge JY, Lemogne C, Hinfray S, Huguet P, Grynszpan O, Tartour E, et al. A meta-analysis of the anterior cingulate contribution to social pain. Soc Cogn Affect Neurosci. 2015 Jan;10(1):19–27.
7. Tan H, Duan Q, Liu Y, Qiao X, Luo S. Does losing money truly hurt? The shared neural bases of monetary loss and pain. Hum Brain Mapp. 2022 Jul;43(10):3153–63.

# Supplementary material 4: Categories of biomarkers investigated by human studies

Data extraction from the retrieved sample of studies by the systematic review identified 297 unique biomarkers. As explained in the method section we develop inductively a list of 14 categories entailing 104 unique biomarkers, leaving the 193 other biomarkers in a 15^th^ category named "Other".

1. “**Organic compounds** (amino-acids, amino-acids derivative, peptides, antioxydants)”: "ascorbate"; "aspartate”; "creatine”; "glutamate”; "glutamine”; "glutathione”; "glycine”; "n-acetylaspartate"; "n-acetylaspartylglutamate"; "taurine"
2. “**Cardiac activity”:** “cardiac deceleration”; “heart rate”; “heart rate variability”
3. **“Cingulate cortex”**: "acc"; "anterior cingulate gyrus"; "anterior middle cingulate cortex"; "cingulate gyrus"; "dorsal acc”; "dorsal posterior cingulate cortex"; "middle acc”; "middle cingulate cortex"; "middle cingulate gyrus"; "perigenual acc"; "posterior cingulate cortex"; "posterior cingulate gyrus"; "posterior middle cingulate cortex"; "pregenual acc"; "rostral acc"; "rostral cingulate cortex"; "subgenual gacc"; "ventral acc"
4. **“Motor frontal areas”: "**premotor area”; “premotor cortex”; “primary motor cortex”; "sma”
5. **“Hippocampus and related areas”**: "hippocampus"; "parahippocampal gyrus"
6. **“Inflammatory or immune response”**: “crp”; “il-1beta”; “il-2”; “il-6” ; “tnf-alpha”
7. **“Insula”**: “anterior insula”; "insula"; “posterior insula”
8. **“Occipital cortex”**: "calcarine”;"cuneus"; "inferior occipital gyrus”; "middle occipital cortex" ; "middle occipital gyrus" ; "occipital cortex" ; "posterior occipital cortex" ; "superior occipital gyrus" ; "visual cortex"
9. **“Parietal lobe”:** "inferior parietal cortex"**;** "inferior parietal gyrus”; "inferior parietal lobule"**;** "medial parietal cortex”; "parietal lobe"**;** "postcentral gyrus"**;** "precuneus"**;** "somatosensory area"; "somatosensory cortex"**;** "superior parietal gyrus"**;** "superior parietal lobule"
10. **“Prefrontal cortex”** (PFC): "apfc"; "caudal middle frontal gyrus"; "dlpfc"; "dmpfc"; "dpfc"; "inferior frontal gyrus"; "inferior ofc"; "lateral orbitofrontal gyrus"; "medial frontal gyrus" ; "medial orbitofrontal cortex"; "middle frontal gyrus"**;** "mpfc”; "ofc"; "pfc"; "precentral gyrus" ; "rostral middle frontal gyrus" ; "superior frontal gyrus"; "superior medial frontal gyrus"; "superior orbitofrontal cortex"; "vlpfc"; "vmpfc"; "vpfc"
11. **“Pupil”:** “pupil dilatation”; “pupil size change”
12. **“Sugars**”: “myo-inositol”; “scyllo-inositol”
13. **“Temporal lobe**”: "inferior temporal gyrus"; "inferior temporal sulcus"; "left midtemporal"; "middle temporal gyrus"; "posterior superior temporal sulcus"; “superior temporal gyrus”; “superior temporal sulcus”; "temporal cortex"; "temporal pole"; “temporal superior gyrus”
14. **“Thalamus”:** “medial thalamus”; “midline thalamus”; “thalamus

*Of note, we categorized the hippocampus and related regions as distinct from other temporal areas. Furthermore, the followings biomarkers were classified in the 'other' category because they overlapped different categories (e.g., concerned multiple brain regions, etc.):*

*“parietal opercular/insular cortex (i.e., s2)"; “temporoparietal junction”; “occipitotemporal cortex”; Groups of brain areas referred to as “network” (e.g., “salience network”, etc.); Groups of brain areas referred to as “cluster” (e.g., "inferior temporal brain cluster", etc.); Lingual gyrus; Fusiform gyrus*

# Supplementary material 5: Biomarkers investigated by human studies

**Table A** presents the most investigated categories of biomarkers, ranked by frequency, according to our inductive classification in 14 categories (Supplementary material 4). **Table B** presents the most investigated biomarkers, ranked by frequency

| **Experimental studies**  **N=68**  **n (%)** | **Observational studies**  **N=24**  **n (%)** | **All studies**  **N=92**  **n (%)** |
| --- | --- | --- |
| **Prefrontal cortex**  36 (52.9) | **Prefrontal cortex**  8 (33.3) | **Prefrontal cortex**  44 (47.8) |
| **Cingulate cortex**  33 (48.5) | **Cardiac activity**  6 (25) | **Cingulate cortex**  37 (40.2) |
| **Insula**  28 (41.2) | **Cingulate cortex**  4 (16.7)  **Inflammatory or immune response**  4 (16.7)  **Parietal areas**  4 (16.7)  **Temporal areas**  4 (16.7) | **Insula**  31 (33.7) |
| **Parietal areas**  16 (23.5) | **Insula**  3 (12.5)  **Thalamus**  3 (12.5) | **Parietal areas**  20 (21.7) |
| **Temporal areas**  12 (17.6) | **Hippocampus and related areas**  2 (8.3)  **Occipital areas**  2 (8.3) | **Temporal areas**  16 (17.4) |
| **Occipital areas**  11 (16.2) | **Organic compounds**  1 (4.2)  **Sugars**  1 (4.2) | **Occipital areas**  13 (14.1) |
| **Thalamus**  9 (13.2) |  | **Thalamus**  12 (13) |
| **Motor frontal areas**  8 (11.8) |  | **Cardiac activity**  9 (9.8)  **Hippocampus and related areas**  9 (9.8) |

**Table A: Most frequent categories of biomarkers investigated by human studies (n=92)** Categories of biomarkers are ranked from the most frequent (first line) to the less. Some studies investigated multiple candidate biomarkers categories with non-physical pain.

| **Experimental studies**  **N=68**  **n (%)** | **Observational studies**  **N=24**  **n (%)** | **All studies**  **N=92**  **n (%)** |
| --- | --- | --- |
| **Dorsal anterior cingulate cortex**  23 (33.8) | **Resting state blood pressure**  4 (16.7)  **Inferior frontal gyrus**  4 (16.7)  **Precuneus**  4 (16.7)  **Superior frontal gyrus**  4 (16.7) | **Dorsal anterior cingulate cortex**  24 (26.1) |
| **Anterior insula**  17 (25.0) | **Anterior cingulate cortex**  3 (12.5)  **Heart rate variability**  3 (12.5)  **Angular gyrus**  3 (12.5)  **Dorso-lateral prefrontal cortex**  3 (12.5)  **CRP** 3 (12.5) **Heart rate** 3 (12.5)  **Heart rate variability** 3 (12.5)  **Supramarginal gyrus** 3 (12.5) | **Anterior insula**  18 (19.6) |
| **Ventro-lateral prefrontal cortex**  16 (23.5) |  | **Ventro-lateral prefrontal cortex**  16 (17.4) |
| **Insula**  12 (17.6) |  | **Insula**  14 (15.2)  **Precuneus**  14 (15.2) |
| **Cerebellum**  10 (14.7)  **Dorso-lateral prefrontal cortex**  10 (14.7)  **Precuneus**  10 (14.7) |  | **Dorso-lateral prefrontal cortex**  13 (14.1) |
| **Inferior frontal gyrus**  8 (11.8)  **Inferior parietal lobule**  8 (11.8) |  | **Inferior frontal gyrus**  **12 (13)** |
| **Anterior cingulate cortex**  7 (10.3)  **Amygdala**  7 (10.3)  **Fusiform gyrus**  7 (10.3)  **Middle temporal gyrus**  7 (10.3)  **Posterior insula**  7 (10.3)  **Superior frontal gyrus**  7 (10.3) |  | **Cerebellum**  11 (12)  **Superior frontal gyrus**  11 (12) |
|  |  | **Anterior cingulate cortex**  10 (10.9) |

**Table B: Most frequent biomarkers investigated by human studies (n=92)**Some studies investigated multiple biomarkers

# Supplementary material 6: Categories of methods of investigation of the biomarkers used in human studies

Data extraction from the retrieved sample of studies by the systematic review identified 28 unique methods of investigation. As explained in the method section, we develop inductively a list of 6 categories entailing 16 unique investigation methods and leaving 12 investigations in the category “Other”.

1. **“Protein expression and kinetic assays”**: "enzyme immunoassays" ; "immunoassay kit"; "kinetic assay kit for amylasis"; "elisa kit"
2. **“Magnetic resonance spectroscopy (MRS)”**: "dpfc levels via proton magnetic resonance spectroscopty" ; " magnetic resonance spectroscopy (mrs)"
3. **“SNP genotyping”**:"snp analysis with pcr-amplification and mass spectrometry"; "snp genotyping method via malfi-tof" ; "snp genotyping with a 5 'nuclease assay"
4. **“EEG”**: "eeg"; "lpp [late positive potential] amplitudes measured via eeg signal after rtms manipulation"; "lpp [late positive potential] amplitudes measured via eeg signal after tms manipulation" ; "theta band activity via intracranial eeg"
5. **“TMS or rTMS”**: "rtms manipulation"; "tms manipulation"
6. **“PET or SPECT”** : "spect"; "mu opioid receptors binding potential via pet imaging with use of a radiotracer"

| **Experimental studies**  **N=68**  **n (%)** | **Observational studies**  **N=24**  **n (%)** | **All studies**  **N=92**  **n (%)** |
| --- | --- | --- |
| **fMRI**  32 (47.1) | **fMRI**  6 (25) | **fMRI** 38 (41.3) |
| **EEG** 14 (20.6) | **Protein expression and kinetic assays**  5 (20.8) | **EEG**  17 (18.5) |
| **TMS or rTMS** 6 (8.8) | **Blood pressure device** 4 (16.7) | **Protein expression and kinetic assays** 10 (10.9) |
| **Protein expression and kinetic assays** 5 (7.4)  **Pupillometry** 5 (7.4) | **EEG**  3 (12.5) | **TMS or rTMS** 6 (6.5)  **Blood pressure device** 6 (6.5) |
| **NIRS** 4 (5.9) | ﻿ **SNP genotyping**  2 (8.3)  **ECG** 2 (8.3) | **ECG** 5 (5.4)  **Pupillometry**  5 (5.4) |
| **ECG** 3 (4.4) | **Magnetic resonance spectroscopty**  1 (4.2)  **PECT or SPECT**  1 (4.2)  **DNA methylation analysis**  1 (4.2)  **Genomic bacterial DNA extraction**  1 (4.2)  **Heart rate monitor paired with wristwatch**  1 (4.2)  **Anatomical MRI**  1 (4.2)  **Peripheral gene expression**  1 (4.2) | **SNP genotyping**  4 (4.3)  **NIRS**  4 (4.3) |
| **SNP genotyping** 2 (2.9) |  | **PECT or SPECT**  3 (3.3) |
| **PET or SPECT** 2 (2.9)  **Blood pressure device** 2 (2.9) |  | **Magnetic resonance spectroscopty**  2 (2.2) |

# Supplementary material 7: Methods of investigation of the biomarkers

**Methods of investigation of the biomarkers used in human studies (n=92), ranked by frequency**

Some studies investigated multiple investigation methods. Investigation methods are grouped by categories and displayed alone if uncategorized.

# Supplementary material 8: Terms and definitions of non-physical pain used in human publications

**Table A** presents the terms used to refer to non-physical pain, ranked by frequency, across the 86 human publications. **Table B** presents the number of publications using 1, 2, 3 and 4 or above different terms to refer to non-physical pain across the 86 human publications. **Table C** present the 22 themes retrieved by the qualitative content analysis of the 36 definitions of non-physical pain identified across the 86 human publications.

| **Experimental publications**  **(N=64)***  **n (%)** | **Observational publications**  **(N=21)***  **n (%)** | **All publications**  **(N=86)**^∆^*****  **n (%)** |
| --- | --- | --- |
| Social pain  55 (85.9) | Psychological pain  15 (71.4) | Social pain  61 (70.9) |
| Social distress  22 (34.4) | Mental pain  6 (28.6) | Psychological pain  25 (29.1) |
| Psychological pain  10 (15.6) | Social pain  5 (23.8) | Social distress  23 (26.7) |
| Painful feelings  8 (12.5) | Emotional pain  4 (19) | Painful feelings  10 (11.6) |
| Hurt feelings  7 (10.9) | Psychache  3 (14.3) | Emotional pain  9 (10.5)  Mental pain  9 (10.5) |
| Emotional pain  5 (7.8) | Spiritual pain  1 (4.8)  Painful feelings  1 (4.8)  Psychological suffering  1 (4.8) |  |
| Mental pain  3 (4.7)  Psychache  3 (4.7) |  | Hurt feelings  8 (9.3) |
|  |  | Psychache  6 (7) |
| Psychic pain  1 (1.6) |  | Spiritual pain 1 (1.2)  Psychic pain  1 (1.2)  Psychological suffering  1 (1.2) |

**Table A: Terms used to refer to non-physical pain across the 86 human publications**

^*^Some publications used different terms

^∆^One publication entailed one observational study and one experimental study: we only counted it in the ‘all publications’ column.

| ***Number of different terms used to refer to non-physical pain within each study*** | **Experimental publications**  **(N=64)**  **n (%)** | **Observational publications**  **(N=21)**  **n (%)** | **All publications (N=86)**^∆^  **n (%)** |
| --- | --- | --- | --- |
| **1** | 25 (29.1) | 11 (12.8) | 36 (41.9) |
| **2** | 31 (36) | 7 (8.1) | 38 (44.2) |
| **3** | 7 (8.1) | 2 (2.3) | 9 (10.5) |
| **4 or above** | 1 (1.2) | 1 (1.2) | 3 (3.5) |

**Table B: Heterogeneity of the terms used to refer to non-physical pain within each human publication (n= 86)** *For instance, 31 experimental publications used 2 different terms to mean refer to a pain not primarily felt in the body*

^∆^One publication entailed one observational study and one experimental study: we only counted it in the ‘all publications’ column.

| ***Themes*** | **All definitions (N=36)**  **n (%)** |
| --- | --- |
| Negative emotion or unpleasant feeling | 32 (88.9) |
| Caused by an objective social disconnection or rejection or exclusion | 25 (69.4) |
| Caused by the subjective perception of being abandoned/rejected | 12 (33.3) |
| Caused by objective social devaluation | 9 (25) |
| Internal response / introspective experience | 7 (19.4) |
| Caused by unmet or frustrated fundamental/core needs | 7 (19.4) |
| Linked/correlated with suicide | 5 (13.9) |
| Unbearable / intolerable | 4 (11.1) |
| Caused by a perception of the self as being deficient | 4 (11.1) |
| Involves same brain regions as physical pain | 3 (8.3) |
| Used terms associated with physical pain | 2 (5.6) |
| Alarm system | 1 (2.8) |
| Leads to mental problems | 1 (2.8) |
| Duration (takes time to resolve) | 1 (2.8) |
| Intensity (can vary quickly) | 1 (2.8) |
| Duration (acute) | 1 (2.8) |
| Experience is similar to physical pain | 1 (2.8) |
| Internal conflict between spiritual beliefs and reality | 1 (2.8) |
| Transcends one biological nature | 1 (2.8) |
| Pain in the soul that is not physical | 1 (2.8) |
| Caused by traumatic memories | 1 (2.8) |
| Motivates to attempt suicide | 1 (2.8) |

**Table C: Thematic contents of the definitions of non-physical pain in human publications (n=86)**

The qualitative content analysis of the 36 definitions of non-physical pain in human publications retrieved 22 themes. *For instance, the theme “Negative emotion or unpleasant feeling” was present in 32 of the definitions (88.9% of the 36 definitions)***.**
